# Tissue-Protective and Anti-Inflammatory Landmark of PRP-Treated Mesenchymal Stromal Cells Secretome for Osteoarthritis

**DOI:** 10.3390/ijms232415908

**Published:** 2022-12-14

**Authors:** Enrico Ragni, Carlotta Perucca Orfei, Paola De Luca, Francesca Libonati, Laura de Girolamo

**Affiliations:** Laboratorio di Biotecnologie Applicate all’Ortopedia, IRCCS Istituto Ortopedico Galeazzi, Via R. Galeazzi 4, I-20161 Milan, Italy

**Keywords:** osteoarthritis, mesenchymal stromal cells, secretome, extracellular vesicles, miRNAs, PRP, regenerative medicine

## Abstract

Bone-marrow-mesenchymal-stromal-cells (BMSCs)- and platelet-rich-plasma (PRP)-based therapies have shown potential for treating osteoarthritis (OA). Recently, the combination of these two approaches was proposed, with results that overcame those observed with the separate treatments, indicating a possible role of PRP in ameliorating BMSCs’ regenerative properties. Since a molecular fingerprint of BMSCs cultivated in the presence of PRP is missing, the aim of this study was to characterize the secretome in terms of soluble factors and extracellular-vesicle (EV)-embedded miRNAs from the perspective of tissues, pathways, and molecules which frame OA pathology. One hundred and five soluble factors and one hundred eighty-four EV-miRNAs were identified in the PRP-treated BMSCs’ secretome, respectively. Several soluble factors were related to the migration of OA-related immune cells, suggesting the capacity of BMSCs to attract lympho-, mono-, and granulocytes and modulate their inflammatory status. Accordingly, several EV-miRNAs had an immunomodulating role at both the single-factor and cell level, together with the ability to target OA-characterizing extracellular-matrix-degrading enzymes and cartilage destruction pathways. Overall, anti-inflammatory and protective signals far exceeded inflammation and destruction cues for cartilage, macrophages, and T cells. This study demonstrates that BMSCs cultivated in the presence of PRP release therapeutic molecules and give molecular ground for the use of this combined and innovative therapy for OA treatment.

## 1. Introduction

Osteoarthritis (OA) is a progressive musculoskeletal condition, estimated to affect 250 million people worldwide [1]. OA is a disease of the whole joint, involving structural alterations in the subchondral bone, ligaments, capsule, synovial membrane, and articular cartilage [2]. Inflammation and recruitment of activated immune cells in the synovial membrane and fluid are crucial OA phenotypes [3]. To date, OA conservative treatments are mainly palliative, while arthroplasty is the definitive treatment [4] for the advanced grade of symptomatic OA. For these reasons, new approaches to manage inflammation and tissue damage in order to delay surgery are actively researched.

The orthobiologics paradigm has emerged in recent years, which refers to the use of biological substances to help musculoskeletal injuries heal more quickly [5]. Their mechanism of action relies on the capacity to interact with pathological resident cells and restore a physiological microenvironment [6]. In this view, orthobiologics treatment may both prevent or delay joint degeneration in early OA grades and also be beneficial in advanced stages, since recent findings have shown that progenitor cells in heavily damaged cartilage respond to biological stimuli [7].

Mesenchymal stromal cells (MSCs) and platelet rich plasma (PRP) are among the most studied orthobiologics. MSCs’ action relies on their paracrine potential, with released cytokines/growth factors and extracellular-vesicle (EV)-embedded miRNAs able to both modulate immune response and promote tissue healing [8]. Accordingly, in recent years, numerous clinical trials using MSCs in OA were started [9], with MSCs resulting as a safe treatment option without serious adverse events, associated with reduced pain, improved joint function, and enhanced overall quality of life. Intriguingly, some studies showed cartilage regeneration after MSC administration, although due to different methods of preparation of these products, the debate concerning the interpretation of MSCs’ regenerative properties is open. Regarding PRP, its mechanism is to enhance the healing cascade through a high concentration of platelets and growth factors [10]. PRP is an available treatment option for OA, and several studies showed beneficial effects for both knee and hip OA in terms of pain, physical functioning, and quality of life [11]. However, cartilage thickness did not improve.

PRP was reported to enhance MSCs’ chondro-inductive properties in vitro [12,13]. In vivo, a combined PRP/MSC intra-articular (IA) injection had a beneficial effect on OA via the stimulation of ECM synthesis and chondrocyte proliferation via the inhibition of the immune reaction [14], with results superior to administration of PRP or MSCs alone. Consistently, studies in humans using the PRP/MSC IA administration showed clinical and functional improvement at the end of follow-up [15,16], with cartilage improvement in MR imaging [17,18]. Similarly, MSCs loaded on platelet-rich fibrin glue, embedded with PRP factors, were applied in full-thickness cartilage defects, with significant improvement in patient functional knee scores and magnetic resonance imaging findings, which were maintained over 12 months postoperatively [19].

To support the combined use of MSCs and PRP for OA, a thorough molecular fingerprint is needed. In this report, soluble mediators and miRNAs embedded in EVs were sifted in the secretome of BMSCs after culture in the presence of PRP levels resembling those cells encounter after IA combined routing. Molecular data were discussed in the frame of OA-related cell types and driving molecules.

## 2. Results

### 2.1. PRP Characterization

PRP hematological data of the 29 patients that were pooled to be used in the study are summarized in Supplementary Table S1. Mean WBC count results were 0.11 × 10^3^/µL ± 0.30, RBC 0.02 × 10^6^/µL ± 0.02, and PLT 333 × 10^3^/µL ± 83. After PRP activation and pooling, ICAM2, PLG, and VCAM1 resulted in the most abundant factors in the liquid fraction (> 100,000 pg/mL) (Supplementary Table S2). The factors laying in the quartile defined by the most abundant molecules are reported in Table 1.

### 2.2. PRP-Treated BMSCs’ Characterization

**Figure 1:**
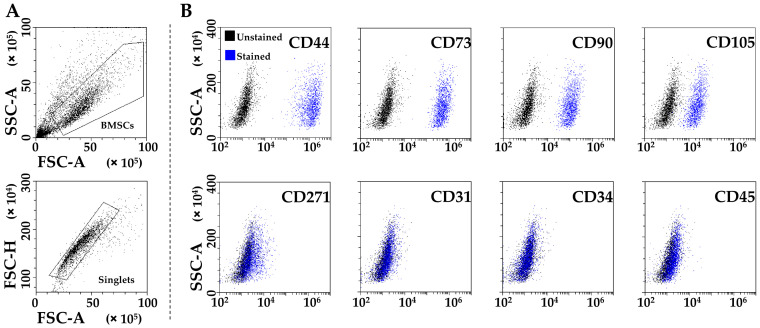
Flow cytometry analysis of PRP-treated BMSCs. (**A**) BMSCs’ identification after debris (upper panel) and aggregate (lower panel) exclusion. (**B**) BMSCs’ staining for general MSC (CD44, CD73, CD90, and CD105, highly positive), BMSC-specific (CD271, dim positivity), and hemato-endothelial (CD31, CD34, and CD45, negative) markers. Representative plots are shown.

PRP-treated BMSCs (from now on called BMSCs for clarity) were strongly positive for MSCs (CD44/73/90/105) and negative for hematoendothelial (CD31/34/45) markers (Figure 1A,B). CD271 was also detected, although at lower levels (24% ± 13 positivity), as previously published for adult MSCs [20].

### 2.3. PRP-BMSCs’ Secreted Factors

**Figure 2:**
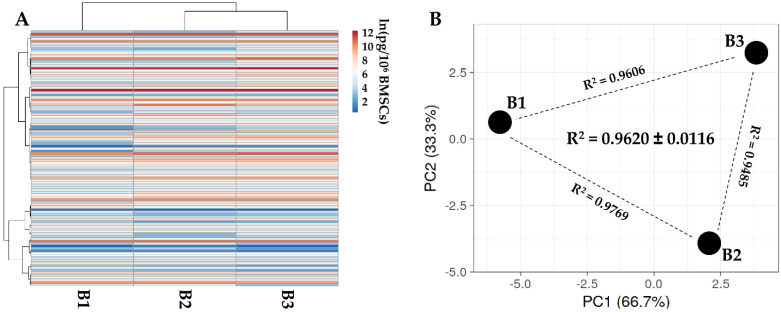
Secreted factor profile comparison between PRP-treated BMSCs under study. A, heat map of hierarchical clustering analysis of the ln(x)-transformed pg/million BMSCs values of detected factors with sample clustering tree at the top. Absolute expression levels reflect the color scale: red shades = high expression levels and blue shades = low expression levels. B, principal component analysis of the ln(x)-transformed pg/million BMSCs values of detected factors. X and Y axis show principal component 1 and principal component 2, which explain 62.5% and 37.5% of the total variance.

One hundred and five soluble factors were identified in all donors (Supplementary Table S3). Hierarchical clustering and PCA analysis samples showed an overall conserved distance (Figure 2A,B), further confirmed by correlation analyses (mean R^2^ of 0.9620 ± 0.0116), allowing the calculation of averaged values for each factor. In Table 2, the most abundant (>10,000 pg/million BMSCs) factors are shown. Two factors had a value of >100,000 pg/million BMSCs, namely IGFBP4 and PF4. Another ten factors resulted between 100,000 and 10,000 pg/million BMSCs, including another two IGFBPs (3 and 6) and two inhibitors of ECM-degrading enzymes (TIMP1 and 2). IGFBP2 is the most abundant in the 10,000 to 1000 pg/million BMSCs group, composed of 28 molecules, including several C-C and C-X-C motif chemokines (CCL5/21/25/26/27 and CXCL11/16). These chemokines also fingerprinted the most represented (37 factors) 1000 to 100 pg/million BMSCs group (CCL1/2/7/8/20/28 and CXCL8/9/10/12), together with several interleukin-related factors (IL1A/6/8/15/16/17B and IL1RN/2RB/6R). IL1B and IFNG laid in the group of the less abundant (<100 pg/million BMSCs) factors (28 members), where, intriguingly, no TNF was again detected, while several TNF receptors and ligands were spread at varying levels of abundance (TNFRSF1A/1B/11B/21 and TNFSF14).

**Figure 3:**
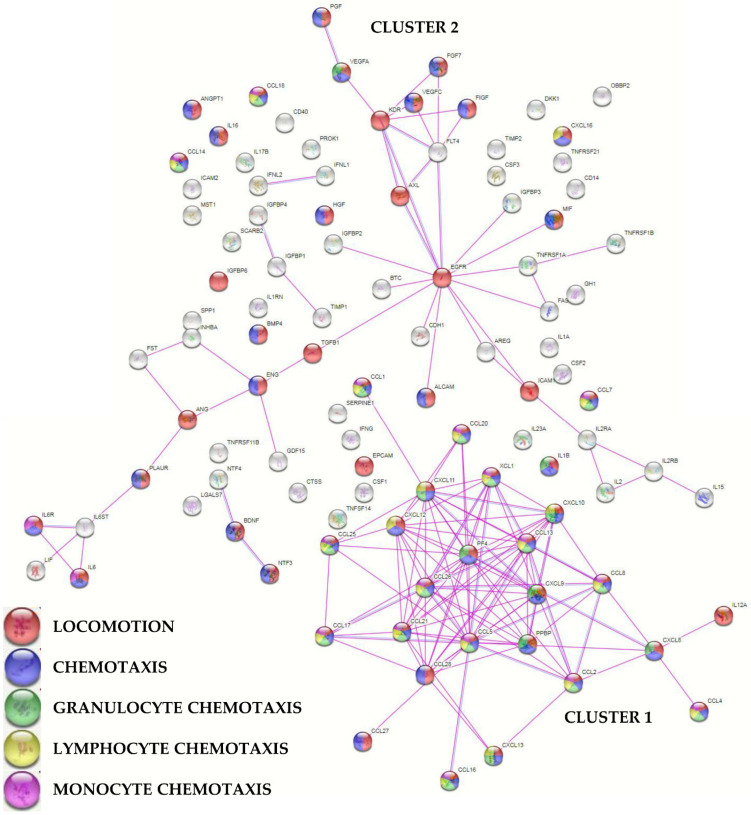
Functional association network for identified PRP-treated BMSCs’ secreted factors. Violet connections are for proteins with experimentally determined interactions; blue connections are for proteins with known interactions based on curated databases. Empty nodes, proteins of unknown 3D structure; filled nodes, known or predicted 3D structure. Locomotion, chemotaxis (with distinction in granulocyte, lymphocyte, and monocyte) GO terms are shown.

To envision the most relevant molecular functions for the detected molecules, a functional protein association network analysis was performed sifting only experimentally validated interactions (Figure 3). One main tight cluster emerged (Cluster 1), composed of 24 proteins, all related to “locomotion” biological process (GO:0040011, Supplementary Table S4 for this term and those following). In particular, 23 players, with the exception of IL12A, were also related to “chemotaxis” (GO:0006935) and 21 with “leukocyte chemotaxis” (GO:0030595). Dissecting the cluster further, 20 factors were related with “granulocyte chemotaxis” (GO:0071621), 17 with “lymphocyte chemotaxis” (GO:0048247), and 13 with “monocyte chemotaxis” (GO:0002548). Another cluster (Cluster 2), spread along the interaction map, was defined by several growth factors, cytokines, and their receptors, with many players again involved in cell movement. In Cluster 2, two small sub clusters emerged, one defined by growth factors and receptors (PGF, VEGFA, VEGFC, FGF7, FIGF, KDR, FLT4, AXL, and EGFR) and another by IL6, together with LIF belonging to the same family of cytokines, and its receptor complex subunits (IL6R and IL6ST). Eventually, a closer look to factors related to extracellular matrix (ECM) homeostasis did not shape a specific cluster, with 12 proteins belonging to the term “extracellular matrix organization” (GO:0030198) and including two ECM protease inhibitors (TIMP1/2).

### 2.4. EVs’ Characterization

**Figure 4:**
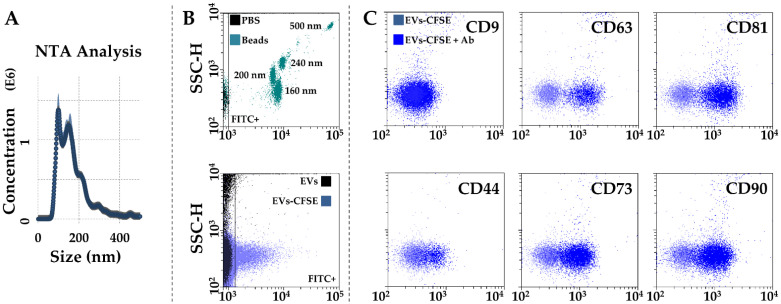
Characterization of PRP-treated BMSC-EVs. (**A**) EVs size analysis from NTA data. (**B**) Flow cytometer calibration to solve FITC-nanobeads, starting from 160 nm (upper panel), and CFSE-labeled EV detection (lower panel) in the FITC channel. (**C**), after gating, CFSE^+^ EVs showed positive staining for CD63 and CD81 EV-defining markers, and CD44, CD73, and CD90 MSC markers. CD9, another EV-postulated marker, was barely detectable. Representative cytograms are presented.

After activated-PRP stimulation, BMSCs released 642 ± 61 EVs per cell in 48 h. The mean EV size calculated by NTA technology resulted 123 nm ± 4, with 73% ± 1 of particles being below 200 nm (Figure 4A). Dimensional analysis was confirmed by flow cytometry through direct comparison with FITC-fluorescent nanobeads, with the majority of EVs falling in the area defined by 160 and 200 nm beads (Figure 4B). Particles were positive for CD63 (91% ± 1) and CD81 (91% ± 0) EV markers, while almost negative for CD9 (4% ± 1), as previously shown in other MSC types [21,22]. CD73 and CD90 MSC markers results were strongly positive (81% ± 1 and 78% ± 7, respectively). CD44, another MSC marker, showed intermediate positivity (44% ± 1), although the complete cloud shift suggests its dim presence in the whole EV population.

### 2.5. EV-miRNAs’ Identification

**Figure 5:**
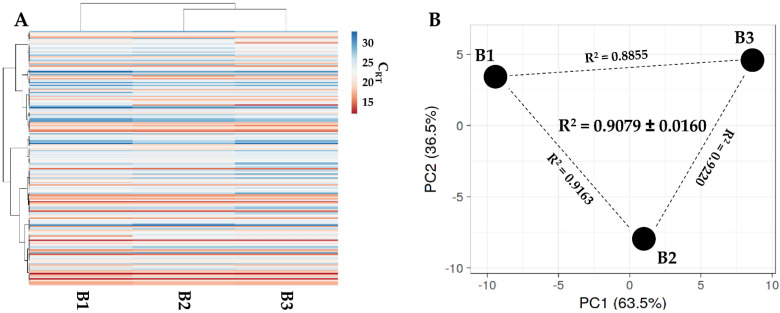
Comparison of EV-miRNAs’ expression profiles between PRP-treated BMSCs under study. (**A**) Heat map of hierarchical clustering analysis of the normalized C_RT_ values of detected miRNAs with sample clustering tree at the top. The absolute expression levels are reflected by the color scale: red shades = high expression levels (low C_RT_ values) and blue shades = low expression levels (high C_RT_ values). (**B**) Principal component analysis of the normalized C_RT_ values of detected miRNAs. X and Y axis show principal component 1 and principal component 2, which explain 63.5% and 36.5% of the total variance.

One hundred and eighty-four miRNAs were detected in all samples in BMSC-derived EVs (Supplementary Table S5). As per secreted factors, hierarchical clustering showed an overall conserved distance (Figure 5A,B), again confirmed by correlation analyses (mean R^2^ of 0.9079 ± 0.0160). This allowed the calculation of average values for each miRNA (Supplementary Table S5). To discuss the most relevant genetic message, it was taken in consideration that in MSC-EVs, no more than one copy per EV is present, even for the most abundant miRNAs [23], and that it was estimated that at least 100 EVs are needed to transfer one miRNA copy to the recipient cell [24]. Thus, only EV-miRNAs in the first quartile of abundance were selected for analysis, ending in 46 molecules covering 96.5% of the identified EV genetic weight (Table 3). The most abundant miRNA turned out to be hsa-miR-24-3p (20.1% of the total genetic weight), followed by hsa-miR-193b-3p (11.4%) and hsa-miR-222-3p (10.1%). The less abundant factors were hsa-let-7e-5p (0.16%), followed by hsa-miR-152-3p (0.15%) and hsa-miR-30a-3p (0.15%). Three miRNAs were excluded from analyses, namely hsa-miR-720 and hsa-miR-1274A/B, since they are likely fragments of tRNA [25]. By sifting experimentally validated miRNA-mRNA interactions (Supplementary Table S6), the miRNAs modulating the highest number of mRNAs were found to be hsa-miR-145-5p (1.49% of total weight, 143 mRNAs), followed by hsa-miR-21-5p (1.09%, 135) and hsa-miR-125-5p (1.73%, 119). Looking at the top three miRNAs, hsa-miR-24-3p (20.12%) regulates 88 mRNAs, hsa-miR-193b-3p (11.35%) 16 mRNAs, and hsa-miR-222-3p (10.08%) 45 mRNAs. Overall, the analyzed miRNAs target 1095 univocal targets (Supplementary Table S7).

### 2.6. OA-Related Targets for PRP-BMSC EV-miRNAs

To predict the impact of the first quartile of EV-miRNAs on OA tissues, univocal mRNA targets were compared to OA regulators expressed in at least 1% of OA chondrocytes, synoviocytes, and different immune cells, including macrophages and T cells [26] (Table 4). Analyzed miRNAs were found to target seven OA cytokines, including IL1A/B and TNF, two of the most studied OA-related pro-inflammatory molecules. In particular, IL1A, targeted by hsa-miR-191-5p (6.73% of the total genetic weight), and TNF, mainly targeted by hsa-miR-125b-5p (1.73%), were the two most regulated molecules. If narrowing the search to factors expressed in less than 1% of tissue cells, IFNG also appeared as heavily targeted by hsa-miR-24-3p (20.12%). Regarding growth factors, first quartile EV-miRNAs may regulate 12 mRNAs, with TGFB1 (main regulator hsa-miR-24-3p, 20.12%) and VEGFA (hsa-miR-320a-3p, 4.20%) being the top modulated factors. In this group, KITLG (hsa-miR-320a-3p, 4.20%), ANGPT2 (hsa-miR-125b-5p, 1.73%), BDNF (hsa-miR-132-3p, 1.01%), TGFB2 (hsa-miR-145-5p, 1.49%), and CTGF (hsa-miR-145-5p, 1.49%) also emerged. Eventually, 10 proteinases or proteinase-related factors with ECM-degrading activity remained, whereas only two ECM-protecting enzymes were targeted. In the first group, MMP14 (mainly by hsa-miR-24-3p, 20.12%), PLAU (hsa-miR-193b-3p, 11.35%), MMP1 (hsa-miR-222-3p, 10.08%), and MMP2 (hsa-miR-125b-5p, 1.73%) could be found. Additionally, MMP-activator APC was targeted at moderate levels (hsa-miR-125b-5p, 1.73%). In the second group, only TIMP3 (hsa-miR-222-3p, 10.08%) was highly regulated. Concerning the single miRNA contribution, hsa-miR-24-3p (20.12%) was found to be the most important one, targeting TGFB1 and MMP14, followed by has-miR-193b-3p (11.35%), targeting the ECM-degrading enzyme PLAU. In third position, hsa-miR-222-3p (10.08%) has a dual role, regulating both MMP1 and TIMP3. Other important EV-miRNAs were found to be has-miR-191-5p (6.73%), targeting pro-inflammatory IL1A, and hsa-miR-320a-3p (4.20%), targeting ECM-catabolism-related VEGFA and synovia inflammation inducer KITLG. Eventually, synovia was found to be the preferential EV-miRNA, targeting all 31 identified factors, followed by chondrocytes and HLA-DR+ immune cells (18 factors each) and T cells (two). Thus, EV-miRNAs are regulators of all OA-affected tissues, with the net balance tipped towards protection.

For the aforementioned reasons, the first quartile of EV-miRNAs was also compared with those reported to be involved at different levels in OA-affected tissues and cells such as cartilage [27], synovia [28], macrophages [29], and T cells [30] (Table 5). For cartilage, eleven miRNAs endorsed with protection and five with destruction were identified, with one player having a dual role. The most expressed (>1% genetic weight) protective miRNAs were hsa-miR-24-3p (20.12%), hsa-miR-193b-3p (11.35%), hsa-miR-222-3p (10.08%), hsa-miR-320a-3p (4.20%), and hsa-miR-125b-5p (1.73%), while their destructive counterparts were hsa-miR-21-5p (1.09%) and hsa-miR-16-5p (1.00%). Thus, overall, protection far exceeded destruction by a 14-fold factor (50.62% vs. 3.61%). The same indication was found also for synovia-related miRNAs, although being at the bottom of this characterization, with only anti-fibrotic has-miR-29a-3p (0.72%) present in the first quartile group. Regarding macrophages, two of the most abundant miRNAs tipped the balance towards the anti-inflammatory M2 phenotype, hsa-miR-24-3p (20.12%) and hsa-miR-222-3p (10.08%), for an overall M2/M1 ratio of 9.6 (30.99% vs. 3.23%). Eventually, we found nine miRNAs linked to T cell activation and only three to its inhibition. Nevertheless, inhibitory the miRNA group included has-miR-24-3p (20.12%), leading to a ratio of 2.9 in favor of T cell inhibition. Thus, overall, protective signals far exceeded OA-driving inputs for all involved tissues.

### 2.7. PRP Effects on BMSC-Secreted Factors and EV-miRNAs

**Figure 6:**
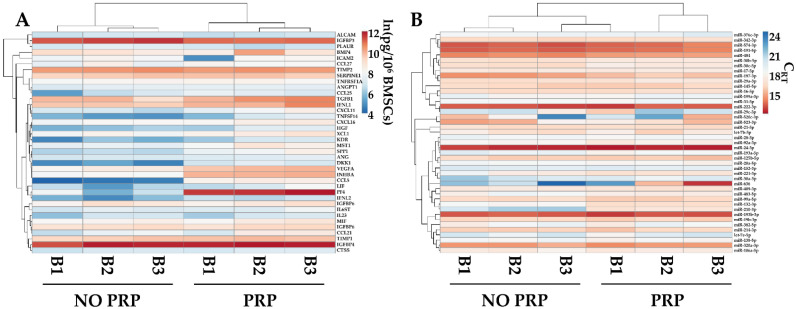
Comparison of abundant PRP-treated BMSCs’ soluble factors (>1 ng/10^6^ cells) and EV-miRNAs (in the first quartile of expression), with counterparts obtained from the same isolates and cultured without PRP (NO PRP). Heat map of hierarchical clustering analysis of the ln(x)-transformed pg/million BMSCs values of detected factors (**A**) and normalized C_RT_ values of detected miRNAs (**B**) with sample clustering tree at the top. The color scale reflects the absolute expression levels: red shades = high expression levels and blue shades = lower expression levels.

The levels of the most abundant factors (>1 ng/10^6^ BMSCs) and EV-embedded miRNAs (first quartile of expression) were compared with data published from our group [31] reporting the secretome of the same BMSCs’ isolates cultured without PRP and analyzed with identical technology. PRP was able to influence the overall pattern of both factors and miRNAs, allowing for the separation of the two conditions, which were found to be sharper for soluble players, as evidenced by hierarchical clustering (Figure 6A,B). Sifting the data in more detail, PRP significantly (*p*-value ≤ 0.05) upregulated PF4 (PRP vs. no-PRP ratio of 177.0 ± 119.1), CCL5 (51.6 ± 7.3), DKK1 (7.4 ± 1.0), and INHBA (6.8 ± 0.6). Regarding miRNAs, PRP upregulated hsa-miR-210-3p (14.9 ± 3.6) and hsa-miR-132-3p (3.1 ± 0.5), while downregulating hsa-miR-197-3p (0.5 ± 0.1).

## 3. Discussion

In this work, the tissue-protective and anti-inflammatory fingerprints of proteins and EV-miRNAs released by PRP-treated BMSCs were identified and characterized in the frame of OA. These data give the molecular basis for the combinatory use of these two biological products for treating tissue degeneration and inflammation in OA patients.

MSCs [32] and PRP [33] showed promising results for the treatment of OA in both clinical trials and everyday practice. Due to these premises, their combined use as an implemented therapy may be of particular interest. As a consequence, few studies showed feasibility for either intra-articular PRP/BMSC administration in OA knees [15,16,17,18] or PRP-derived fibrin glue/BMSC transplantation into OA cartilage defects [19]. In all cases, authors reported clinical and functional amelioration, with significant cartilage improvements noted on MR imaging follow-up in two IA studies [17,18] and when MSCs were loaded onto PRP gels. Nevertheless, to date and to our knowledge, no preclinical or clinical data have been published for the molecular signals delivered by MSCs in the presence of PRP at concentrations similar to what those cells may encounter during the combinatorial therapy, and that may explain the positive outcomes and give ground for a wider use of the combined approach. This study was aimed at filling this gap.

Secreted factors analysis showed the presence of several cytokines and chemokines involved in leukocyte migration and chemotaxis. This is of relevance, since it is postulated that MSCs may better regulate immune cells’ functions when in close proximity. In this frame, one of the most powerful molecules is IL1RN, also known as IL1RA (Interleukin 1 Receptor Antagonist), found at 500 pg/mL concentration in our ELISA assays. IL1RA can promote the polarization of macrophages toward the anti-inflammatory M2 phenotype [34], which secrete high levels of IL10 and low levels of TNF and IL17. This modulation in anti/pro-inflammatory cytokines balance may be further enhanced by MSC-secreted IL6 and hepatocyte growth factor (HGF) [35], both found in this study at 500 and 1500 pg/mL. Moreover, M2 macrophages may induce the formation of FoxP3^+^ Tregs from naïve CD4^+^ T cells through the release of CCL18 and TGFB1 [36,37], which are also found in analyzed secretomes at 50 and 40,000 pg/mL, respectively, thus contributing to the macrophage-induced formation of Treg. Consistently, the neutralization of CCL18 and TGFB1 leads to a significant reduction in MSC-induced Treg formation [36,38] from conventional T cells. Moreover, when in proximity, MSCs suppress T cell proliferation [39] and activation [40]. Interestingly, TGFB and HGF were identified among the soluble mediators of BMSCs’ anti-proliferative effects [41], and cellular or soluble ICAMs (ICAM1 at 200 and ICAM2 at 2900 pg/mL in the secretome) that are some of the molecules responsible for T cell activation suppression [42]. MSCs also cause a shift from pro-inflammatory Th1 to anti-inflammatory Th2 cells with a change in their cytokine profile toward anti-inflammation [43,44], with the secretion of IL4 in Th2 cells and the decrease in the IFNG production by Th1 cells. Eventually, MSCs reduce tissue damage given by activated neutrophils [45]. Therefore, PRP-treated BMSCs secrete an array of molecules to attract all immune cells involved in OA in order to create a gradient in the local concentration of factors able to promote a shift towards anti-inflammatory phenotypes. This is a crucial component of the inflammation reduction observed after combined PRP/MSC therapy.

PRP-treated BMSC secretomes also contain high levels of factors involved with ECM homeostasis, a crucial OA feature. In fact, during OA, ECM of cartilage is actively remodeled by an altered balance between, among others, matrix-degrading proteins, including matrix metalloproteinases (MMPs) and their inhibitors (TIMPs) [46]. Of note, TIMP1 and 2 were among the four factors with an ECM-related role laying in the 10 most abundant detected proteins. This is of relevance, since TIMPs are envisioned as a potential therapeutic modality for decreasing the detrimental effects of MMPs through direct inhibition. Accordingly, TIMP1/2 supplementation completely prevented the release of collagen fragments from ex vivo bovine cartilage [47], and approaches aimed at rebalancing the TIMP/MMP ratio are actively sifted [48]. Similarly, supplementation with TGFB1, the most abundant ECM-related factor in the secretome, was also proposed as an OA therapy [49], since TGFB1 is involved in cartilage homeostasis at different levels [50]. Nevertheless, its increase in the synovial fluid of OA patients suggests that a tight control of its abundance and activation at the cartilage and chondrocyte levels may turn beneficial effects into detrimental cartilage phenotypes [51]. The other abundant and ECM-related secreted factor is Serpine1. Serpine1 inhibits urokinase and tissue-type plasminogen activators [52] that turn plasminogen into the active form plasmin, which is able to degrade the ECM directly by the cleavage of components such as fibronectin, glycoproteins, and proteoglycans and to activate numerous MMPs [53]. Interestingly, in OA, Serpine1 levels are decreased, suggesting an increased proteolytic load [54]. Eventually, even if not directly associated with ECM, abundantly secreted insulin-like growth factor (IGF) binding proteins (IGFBP2/3/4/6) may regulate matrix homeostasis. In fact, IGF1 is the crucial factor in synovial fluid that promotes cartilage matrix anabolism [55]. Its binding to IGFBPs may alter its bioavailability, ending in still-controversial action on cartilage and chondrocytes. On one hand, IGFBP-bound IGF1 is unable to act on its specific cell receptors; on the other hand, macromolecular complexes between IGFBS and IGF1 are degraded during OA, leading to increased amounts of available IGF1 around chondrocytes [56]. Eventually, BMP4, expressed at >10,000 pg/mL, promotes cartilage growth, matrix deposition, and chondrocyte proliferation [57], and its levels are reduced in OA [58]; therefore, it is envisioned as a therapeutic molecule for OA [59]. Therefore, most abundant PRP-treated BMSCs’ secreted factors have a modulatory effect on ECM homeostasis, being a pillar of the molecular explanation for the observed cartilage protection observed in the PRP/MSCs combined therapy.

Together with soluble factors, several EV-embedded miRNAs were also found to be able to target inflammation and ECM-related molecules and almost all cell types involved in OA. Within the cytokines most frequently associated with OA, IL1A, IL1B, and TNF were all EV-miRNA-targeted. These data confirm the combined PRP/BMSC strategy as a new therapeutic option to target pro-inflammatory cytokines, since the results of clinical trials of therapeutic candidates such as Abs or blocking molecules have been rather unsatisfactory for both IL1A/B [60,61,62] and TNF [63,64]. In this frame, RNA-based therapeutics have been proposed as cutting-edge options, with advanced lipid nanoparticle formulations, such as natural EVs, dramatically improving both the stability and delivery of RNA molecules. The same paradigm can be envisioned also to target matrix-degrading enzymes. EV-miRNAs target 10 factors directly (mainly MMPs, ADAMs, and ADAMTs) or indirectly degrading ECM, and only two TIMPs. As for pro-inflammatory cytokines, ECM-degrading enzymes targeting miRNAs in EVs may be new biologic- and RNA-based therapeutics overcoming the difficulties observed for MMP inhibitors that, although having shown notable effects on preclinical OA models, only in rare cases have entered clinical trials for patients with knee OA [65]. An advantage of EV-miRNAs might be the route of PRP/BMSC administration, which is intra-articular and therefore localized to the damaged tissues. In fact, general MMP inhibitors may broadly affect the matrix turnover in musculoskeletal tissues other than cartilage, making them unsuitable for OA treatment due to possible side effects [66]. Regarding growth factors, TGFB1 and VEGFA are the most targeted. As previously discussed, TGFB1 is highly abundant as a secretome-soluble factor. If on one side this might be of crucial importance to improve cartilage synthesis, on the other side its excess may result in side effects, such as fibrosis and osteophyte formation [49]. Therefore, the dual TGFB1 regulation relying on a sudden release and a subsequent inhibition in its production by all OA-involved cell types might be beneficial. This is consistent with the proposed idea, as therapeutics, of the exogenous supplementation of TGFB1 balanced by its local inhibition, as EV-miRNAs can do. A similar paradigm can be envisioned also for VEGFA, highly abundant in the secretome. The reduction in its de novo secretion may reduce its total levels, which, when too high, are significantly correlated with OA and greater vascular invasion into articular cartilage [67]. Overall, the majority of targeted growth factors are involved in angiogenesis, osteophyte formation, and cartilage catabolism, with few exceptions, such as IGF1 and 2, which are involved in ECM anabolism. Therefore, EV-miRNAs are another pillar for the observed anti-inflammatory and regenerative features of PRP-combined BMSCs.

Together with single OA-related molecule modulation, EV-miRNAs have been found to regulate OA tissues and cell types on a general level. For synovia, the paucity of literature-reported OA-related miRNAs renders a clear prediction weak, although almost all EV-miRNA-targeted factors are expressed by synoviocytes. For cartilage, macrophages, and T cells, most abundant EV-miRNAs have regenerative and anti-inflammatory properties. These features mainly rely on three miRNAs, hsa-miR-24-3p (20.12% of the total genetic weight), hsa-miR-193b-3p (11.35%), and hsa-miR-222-3p (10.08%). Regarding cartilage, all three miRNAs are involved as the main contributors for the high protection/degeneration ratio (14-fold). hsa-miR-24-3p levels are reduced in OA patients and chondrocytes, where its overexpression reduced cell injury and increased viability [68]. Similarly, hsa-miR193b-3p is reduced in OA cartilage [69], and its overexpression leads to aggrecan synthesis [70] in vitro and strongly enhanced cartilage formation in vivo [69]. Again, has-miR-222-3p is downregulated in OA cartilage [71] and chondrocytes [72], and its overexpression into the cartilage of medial meniscus destabilized mice significantly reduced cartilage destruction [72]. Regarding macrophages, hsa-miR-24-3p and hsa-miR-222-3p are the main contributors for the induction of the anti-inflammatory phenotype. hsa-miR-24-3p is a crucial player in the lifespan of all monocytes and macrophages. It is necessary for the acquisition of Mφ-specific functionality from monocytes and, when overexpressed, reduces the release of inflammatory cytokines and increases the amount of the M2 marker CD206 [73]. Consistently, in mature macrophages, hsa-miR-24-3p overexpression decreased the production of M1 phenotype markers, whereas it increased the production of M2 markers, with an opposite effect observed upon its knockdown [74]. hsa-miR-222-3p is elevated during monocytic cell differentiation toward macrophages [75] and is an M2-responsive miRNA [76]. Of note, as may happen with PRP-BMSCs’ EVs acting on joint and synovia-resident macrophages, epithelial-ovarian-cancer-cell-derived EVs are able to induce an M2 phenotype through the transfer of highly abundant embedded hsa-miR-222-3p [77]. Eventually, in the frame of T cells, hsa-miR-24-3p is again the main actor to counteract activation, since it may repress IFNG production in both CD4^+^ [78] and CD8^+^ [79] lymphocytes. Moreover, as previously discussed for enriched hsa-miR-222-3p in tumor cell EVs, cancer patients’ EVs enriched in miR-24-3p inhibited T cell proliferation and Th1 and Th17 differentiation and induced Treg differentiation [80]. Thus, the presence of few abundant miRNAs with high anti-inflammatory and tissue-protective features again supports on a molecular basis the results observed in the clinical setting for PRP-treated BMSCs and offers opportunities for the development of EV-based therapies relying on the presence of a few highly committed therapeutic miRNAs.

Eventually, PRP demonstrated to be able to modulate the secretory fingerprint of BMSCs, although in the frame of a high conservation of the overall message. In fact, despite the clear distinction observed in the heat maps for secreted factors and EV-miRNAs with respect to untreated cells, only a few molecules were found to be statistically different without the involvement of the most disease-therapeutic players. PF4 was the most modulated protein (177-fold). Being this factor amongst the most abundant in PRP, the observed result might be due to its incorporation into BMSCs during culturing and subsequent release during starvation. Nevertheless, the absence of such a conserved pattern of uptake and release for other PRP-enriched molecules suggests a possible upregulation of its production and excludes residual PRP contamination during starvation. The second most upregulated protein was CCL5 (51.6-fold), found to be elevated in OA patients’ synovial fluid and postulated to promote IL6 production in synoviocytes [81]. This possible detrimental effect was balanced by the upregulation of DKK1 (7.4-fold) and INHBA (6.8 fold), having a protective effect by preventing cell hypertrophy in chondrocyte terminal differentiation [82] and inhibiting aggrecanase-mediated cleavage of cartilage [83], respectively. Regarding EV-miRNAs, hsa-miR-210-3p was the most increased (14.9-fold). It was demonstrated to have several protective effects, including inhibition of subchondral angiogenesis [84], enhancement of chondrogenic differentiation [85], and reduction in inflammation in the articular cavity in OA rats [86]. The second upregulated miRNA was hsa-miR-132-3p (3.1-fold), which was found to be decreased in OA patients [87], with its overexpression leading to elevated chondrocyte homeostasis and decreased inflammation, possibly by the targeting of MMP9 and BDNF as emerged by in silico analysis. Finally, hsa-miR-197-3p was mildly downregulated (2-fold) after PRP treatment. This molecule was shown to be downregulated in OA cartilage and suggested to have a protective effect on cartilage and inflammation [88]. Therefore, these fluctuations in both protective and damaging factors suggest that high levels of PRP do not interfere with the BMSCs’ overall secretome potential. This is of relevance, since it was postulated for adipose-derived MSCs that high (40 to 60%) PRP levels could alter cell phenotypes with respect to lower concentrations (up to 20%) [89]. In fact, in OA joints, PRP is usually administered with volumes (5–10 mL) similar to those of the synovial fluid that is sometimes aspirated before the injection, ending in a 50%, or even higher, final concentration, which is close to what was used in our experimental setting. Moreover, when a PRP clot is used as a scaffold for BMSCs, the levels of factors locally released around the cells are presumably more elevated than those in a 10–20% PRP culture. Thus, overall, the BMSCs’ secretome fingerprint observed in this study with high PRP concentrations is of relevance for the use of the BMSCs/PRP combined clinical application for OA, and its conservation with respect to untreated cells suggests that the improved healing capacity observed for the joined treatment might rely on an addictive effect rather than BMSCs’ potentiation in the presence of PRP. Moreover, due to the turnover of synovial fluid in the joints, it is possible that some release factors or EVs might reach the bloodstream and lymphatic system. To date, no adverse effects have been reported in patients that received MSC-sourced secretome, indicating that the local and systemic presence of MSC secretome or MSC-EVs is safe [90]. Moreover, even if from different sources, MSC secretome administered intravenously was able to both recover cartilage, with a reduction in inflammation, and reduce neuropathic pain in in vivo models of arthritis [91] and osteoarthritis [92], respectively. Therefore, the “leakage” of secretome components into the bloodstream could be an additional extra weapon to treat joint damage. In this frame, the presence of BMSCs will guarantee the replacement of cleared molecules and EVs, at least for the first days after treatment, suggesting that a single administration may be the most applicable protocol in the majority of registered clinical trials for musculoskeletal diseases, while in the only two trials using the secretome, more than one administration is proposed (NCT04314661 and NCT05579665), due to the lack of a continuous release of factors and nanoparticles.

The present study has some limitations. We are aware that the number of donors is limited. Nevertheless, the consistency of soluble factors and EV-miRNAs’ amount confirmed by correlation analyses across the donors suggest a conserved message for the clinical efficacy of the PRP-BMSC therapy. An issue could be the stringent selection we applied for the donors used in this study, which were all females with a comparable age range. Thus, the results herein presented will need to be validated in the future in male donors and, in general, in donors with a wider age range. We are also aware that the number of analyzed molecules, for both soluble factors and miRNAs, is limited. We preferred to focus our attention on very well-characterized factors, with the characterization of a larger portfolio of molecules to date poorly described, especially for miRNAs that in the last release of miRBase are close to 40,000 units, being the goal of future studies. Another limitation is that BMSCs, even when co-administered with PRP in the joint space, are in direct contact with synovial fluid and other tissues, which may alter factors and EVs’ release. Moreover, the BMSC-to-PRP ratio is another issue. In our experiments, this value was 1.2 × 10^6^ ± 0.2/mL, having used PRP at a high concentration, 50% of the total cell culture volume, to mimic the situation BMSCs encounter after intra-articular administration with PRP. This value was selected, since in our hospital, 5 mL of PRP are usually injected into the joint space, and in clinical trials a number of 1 to 100 × 10^6^ BMSCs are reported to be administered, with 1 to 10 × 10^6^ cells being the preferred option. Thus, it is reasonable to consider a BMSC-to-PRP ratio around 1 as a recommended option, although other ratios have to be tested in the future to optimize the best choice for clinical applications. Eventually, the system we used to produce the secretome relies on FBS deprivation during starvation, again possibly altering the physiological release of molecules and the BMSCs’ metabolism. At least for EVs, further studies with EV-depleted FBS or human platelet lysates as an energy source may be envisioned since, to date, a sharp removal of microparticles from serum is far to be clearly defined.

## 4. Materials and Methods

### 4.1. PRP Collection, Activation and Storage

An aliquot (1300 µL ± 700) of PRP obtained with the Endoret^®^ system (BTI, Vitoria, Álava, Spain) was collected from 29 consecutive patients (12 females, 17 males, mean age 56 ± 16 years) undergoing PRP-based regenerative orthopedic procedures at RE.GA.IN.^®^ Center of IRCCS Istituto Ortopedico Galeazzi. PRP cell content was assessed with a Sysmex XN-2000 hemocytometer (Sysmex, Kobe, Japan). After PRP activation (22.8 mM CaCl_2_, 2 h at 37 °C), clots were removed by centrifugation (2800× *g*, 15 min at RT) and supernatants stored at −80 °C. Before experiments were performed, single aliquots were pooled.

### 4.2. ELISA Characterization of Activated PRP

Two hundred fifty microliters of pooled PRP were two-fold diluted before secreted factors detection with the enzyme-linked immunosorbent assay (ELISA) Quantibody^®^ Human Cytokine Array 4000 Kit (RayBiotech, Norcross, GA, USA, https://www.raybiotech.com/quantibody-human-cytokine-array-4000/, accessed on 16 May 2022) following the manufacturer’s protocol and four technical replicates. Concentrations were determined by comparison with standard samples.

### 4.3. Bone Marrow Collection, BMSCs’ Isolation and Expansion

Bone marrow was collected from 3 female donors with mean age 50 ± 2 years. Fifty thousand nucleated cells per cm^2^ were seeded in αMEM (Thermo Fisher Scientific, Waltham, MA, USA) supplemented with 10% FBS at 37 °C, 5% CO_2_, and 95% humidity. After 3 days, new medium was added, and BMSCs were selected for plastic adhesion. After 2 weeks, colonies were detached, BMSCs were seeded at 4000/cm^2^, and cells were cultured up to passage 3. Before secretome collection, BMSCs at 90% confluence were cultured for 2 days in the presence of 50% pooled PRP (1:1 diluted in complete cell culture medium, for a BMSCs to PRP ratio of approximately 1.2 × 10^6^ ± 0.2/mL), then washed 3 times with PBS, and finally, serum-free αMEM was added at a ratio of 0.07 mL/cm^2^. After 48 h, the secretome was collected and centrifuged at 376× *g* for 5 min at 4 °C, 1000× *g* for 15 min at 4 °C, 2000× *g* for 15 min at 4 °C, and twice at 4000× *g* for 15 min at 4 °C. Clarified secretomes were used for EVs’ and ELISA analyses. After secretome removal, BMSCs were counted and viability was assessed with a NucleoCounter NC-3000 (ChemoMetec, Allerod, Denmark).

### 4.4. Flow Cytometry Characterization of PRP-Treated BMSCs

BMSCs at passage 3 after 2 days in 50% pooled PRP were detached and stained for 30 min at 4 °C in the dark with both hemato/endothelial (CD31-PerCP Vio700 clone REA730, CD34-FITC clone AC136, CD45-PE Vio770 clone REA747) and MSC (CD44-PE Vio770 clone REA690, CD73-PE clone REA804, CD90-FITC clone REA897, CD105-PerCP Vio700 clone REA794, CD271-PE clone REA844) markers (Miltenyi Biotec, Bergisch Gladbach, Germany). After washing with FACS buffer, BMSCs were detected by flow cytometry with a CytoFLEX flow cytometer (Beckman Coulter, Fullerton, CA, USA), collecting a minimum of 30,000 events. The following combinations of antibodies were used: CD34/271/31/45 and CD73/90/105/44.

### 4.5. ELISA Characterization of PRP-Treated BMSCs Secretome

Two-hundred-fifty-microliter secretomes from PRP-treated BMSCs were two-fold diluted before secreted factors detection with the enzyme-linked immunosorbent assay (ELISA) Quantibody^®^ Human Cytokine Array 4000 Kit, as previously described. The amount of each factor in pg/mL was converted into pg/million cells by multiplying the original value for the total collected volume in mL and finally dividing by the total number of cells. Values are shown as mean ± SD.

### 4.6. Protein–Protein Interaction Networks

Interactome maps of ELISA-identified proteins were generated with the STRING tool (http://www.string-db.org, accessed on 12 May 2022) (database v11.5). The following properties were selected: (i) organism, Homo sapiens; (ii) meaning of network edges, evidence; (iii) active interaction sources, experiments; (iv) minimum required interaction scores, low confidence (0.150).

### 4.7. Characterization of EVs in PRP-Treated BMSCs’ Secretomes

Flow cytometry: cleared secretomes were 1:1 diluted with PBS and divided into 3 aliquots: (i) unstained, (ii) 5(6)-carboxyfluorescein-diacetate-succinimidyl-ester (CFDA-SE, Sigma-Aldrich, St. Louis, MO, USA)-stained (1 µM final concentration, 30 min at 37 °C), (iii) after CFDA-SE supplementation and incorporation leading to FITC-fluorescent carboxyfluorescein succinimidyl ester (CFSE), CD9-APC clone HI9A, CD63-APC clone H5C6, CD81-APC clone 5A6, CD44-APC clone BJ18, CD73-APC clone AD2, CD90-APC clone 5E10 (Biolegend, San Diego, CA, USA) stained (30 min at 4 °C). After a further 1:3 dilution with PBS, samples were analyzed with a CytoFlex flow cytometer. At least 30,000 events were collected. FITC-fluorescent nanobeads of 160, 200, 240, and 500 nm (Biocytex, Marseille, France) were used as internal control.

Nanoparticle tracking analysis (NTA): cleared secretomes were 1:1 diluted in PBS and visualized by Nanosight NS-300 system (NanoSight Ltd., Amesbury, UK) (5 recordings of 60 s). NTA software v3.4 provided both concentration measurements and high-resolution particle size distribution profiles.

### 4.8. Total RNA Isolation from EVs’ and miRNAs’ Quantification

Five milliliters of cleared secretomes were 1:1 diluted in PBS and ultracentrifugated (100,000× *g*, 9 h at 4 °C). After addition of exogenous *Arabidopsis thaliana* ath-miR-159a (30 pg) synthetic miRNA spike to evaluate RNA recovery and cDNA synthesis, total RNA was extracted with miRNeasy and RNeasy Cleanup Kits (Qiagen, Hilden, Germany), as previously reported [93]. The expression of 754 miRNAs was evaluated with the OpenArray system (Life Technologies, Foster City, CA, USA) with 384-well OpenArray plates, according to the manufacturer’s instructions. Each single miRNA was considered as present and considered for further analyses only when amplification appeared in all three samples. ath-miR-159 spike-in C_RT_ was used for the equalization of technical differences. Eventually, the global mean method [93] allowed the normalization between samples.

### 4.9. Identification of miRNAs’ Target

miRTarBase v8.0 was used to identify targets of identified miRNAs (https://mirtarbase.cuhk.edu.cn/~miRTarBase/miRTarBase_2022/php/index.php, accessed on 14 March 2022) [94]. Only miRNA-mRNA interactions supported by strong experimental evidence were considered.

### 4.10. Comparison of PRP-Treated vs. Untreated BMSCs’ Secretomes

Soluble factors and EV-miRNAs’ expression data from BMSCs cultivated in the absence of PRP were retrieved from a publication from our group [31]. Donors, BMSCs’ passage, culturing conditions (except PRP treatment), and technical platforms for ELISA and qRT-PCR were identical to those used in this study. For miRNA comparison, the global mean normalization method on the whole dataset for molecules detected in both conditions was used.

### 4.11. Statistical and Computational Analyses

GraphPad Prism Software v8 (GraphPad, San Diego, CA, USA) was used for statistical analyses. For factors’ and miRNAs’ comparison between PRP and non-PRP samples, normal data distribution was assessed by Shapiro–Wilk normality test (α of 0.05). When the normality test was passed, a parametric t-test was performed. When the normality test was not passed, a nonparametric t-test was executed. The level of significance was set at *p*-value ≤ 0.05. The linear association between samples was estimated with the Pearson correlation coefficient (R^2^) formula. The results were interpreted according to the degree of association [95].

Hierarchical clustering and principal component analysis (PCA) were conducted with the ClustVis package (https://biit.cs.ut.ee/clustvis/, accessed on 16 May 2022) [96]. Maps were generated using the following settings for both rows and columns clustering distance and method: correlation and average, respectively.

## 5. Conclusions

Secreted factors and EV-miRNAs account for the anti-inflammatory and tissue-regenerative properties of BMSCs when administered to OA joints in combination with PRP. Several soluble factors were found to be associated with the migration of OA-related immune cells, suggesting the ability of BMSCs to attract lympho-, mono-, and granulocytes and eventually modulate their homeostasis and inflammatory status. In this frame, several EV-miRNAs had an immunomodulating role at both the single-factor and cell level, alongside the capacity to target OA-characterizing ECM-degrading enzymes and cartilage destruction pathways. Overall, anti-inflammatory and protective signals far exceeded inflammation and destruction cues for cartilage and immune cells, such as macrophages and T cells. This study gives molecular ground for the use of the PRP-BMSCs’ combined therapy for OA and, after molecular sifting for the identification of disease-specific therapeutic miRNAs, for other musculoskeletal diseases where tissue degeneration and inflammation are pathological landmarks.

## Figures and Tables

**Table 1:** Most abundant soluble factors in pooled activated PRP.

TYPE	FACTOR	(pg/mL)	
CYT	ICAM2	790,983	Intercellular adhesion molecule 2
CYT	PLG	182,992	Plasminogen
REC	VCAM1	102,461	Vascular cell adhesion protein 1
CYT	IL13RA2	94,449	Interleukin-13 receptor subunit alpha-2
CHE	IL9	73,686	Interleukin-9
GF	IGFBP4	72,637	Insulin-like growth factor binding protein 4
REC	MICB	66,893	MHC class I polypeptide-related sequence B
GF	TGFB1	58,819	Transforming growth factor beta-1
CHE	PF4	56,183	Platelet factor 4
CYT	IL23A	47,225	Interleukin-23 subunit alpha
CYT	FLT1	47,188	Vascular endothelial growth factor receptor 1
INF	PDGFB	47,171	Platelet-derived growth factor subunit B
CHE	IL31	38,293	Interleukin-31
GF	BMP7	35,835	Bone morphogenetic protein 7
CYT	SIGLEC5	35,491	Sialic-acid-binding Ig-like lectin 5
GF	IGFBP3	34,608	Insulin-like growth factor binding protein 3
CHE	CCL26	34,511	C-C motif chemokine 26
GF	FGF4	33,301	Fibroblast growth factor 4
CHE	SPP1	30,604	Osteopontin
GF	BMP4	28,461	Bone morphogenetic protein 4
GF	IGFBP2	27,669	Insulin-like growth factor binding protein 2
REC	IL21R	26,521	Interleukin-21 receptor
REC	SELL	25,729	L-selectin
GF	CSF1R	24,163	Macrophage-colony-stimulating factor 1 receptor
CYT	IL6ST	23,811	Interleukin-6 receptor subunit beta
INF	IL6R	23,412	Interleukin-6 receptor subunit alpha
CHE	CCL21	23,105	C-C motif chemokine 21
INF	TIMP2	22,795	Metalloproteinase inhibitor 2
REC	TNFRSF17	22,355	Tumor necrosis factor receptor superfamily member 17
CHE	IFNL1	19,506	Interferon lambda-1
CYT	CDH1	17,919	Cadherin-1
CYT	IL13RA1	16,766	Interleukin-13 receptor subunit alpha-1
GF	KDR	15,881	Vascular endothelial growth factor receptor 2
CYT	TREM1	15,662	Triggering receptor expressed on myeloid cells 1
CYT	CTSS	15,538	Cathepsin S
REC	TNFRSF18	13,410	Tumor necrosis factor receptor superfamily member 18
CYT	LGALS7	12,956	Galectin-7
CYT	TNFRSF10D	12,579	Tumor necrosis factor receptor superfamily member 10D
CYT	PDGFA	12,454	Platelet-derived growth factor subunit A
INF	TNFRSF1A	12,348	Tumor necrosis factor receptor superfamily member 1A
GF	TGFA	12,273	Protransforming growth factor alpha
INF	TIMP1	12,041	Metalloproteinase inhibitor 1
GF	FGF7	11,984	Fibroblast growth factor 7
CYT	IL2RB	11,830	Interleukin-2 receptor subunit beta
CYT	SERPINE1	11,194	Plasminogen activator inhibitor 1
CHE	CXCL16	10,931	C-X-C motif chemokine 16
INF	ICAM1	10,930	Intercellular adhesion molecule 1
INF	CCL5	10,751	C-C motif chemokine 5
INF	TNFRSF1B	10,501	Tumor necrosis factor receptor superfamily member 1B

CHE = chemokine; CYT = cytokine; GF = growth factor; INF = inflammation; REC = receptor.

**Table 2:** Most abundant (>10,000 pg/million cells) secreted factors in PRP-treated BMSCs.

		pg/Million Cells					
TYPE	FACTOR	B1	B2	B3	MEAN	SD	
GF	IGFBP4	146,361	166,965	160,221	157,849	8577	Insulin-like growth factor binding protein 4
CHE	PF4	118,491	127,082	177,384	140,985	25,975	Platelet factor 4
GF	IGFBP3	67,919	65,875	69,956	67,917	1666	Insulin-like growth factor binding protein 3
GF	TGFB1	25,432	43,973	51,332	40,245	10,897	Transforming growth factor beta-1
INF	TIMP2	37,691	43,548	37,878	39,706	2718	Metalloproteinase inhibitor 2
CHE	IFNL1	25,976	26,330	49,299	33,869	10,912	Interferon lambda-1
CYT	INHBA	25,821	24,754	27,428	26,001	1099	Inhibin beta A chain
GF	VEGFA	18,261	24,211	24,511	22,328	2878	Vascular endothelial growth factor A
INF	TIMP1	19,604	23,458	20,658	21,240	1626	Metalloproteinase inhibitor 1
CYT	SERPINE1	20,131	20,479	16,545	19,052	1778	Plasminogen activator inhibitor 1
GF	BMP4	6102	33,281	8863	16,082	12,213	Bone morphogenetic protein 4
GF	IGFBP6	9679	14,168	13,933	12,593	2063	Insulin-like growth factor binding protein 6

CHE = chemokine; CYT = cytokine; GF = growth factor; INF = inflammation.

**Table 3:** EV-miRNAs falling in the first quartile of abundance.

	C_RT_					
miRBase ID	B1	B2	B3	Mean	SD	% Weight
hsa-miR-24-3p	12.24	12.53	12.40	12.39	0.12	20.12142652
hsa-miR-193b-3p	12.96	13.40	13.28	13.21	0.18	11.35032031
hsa-miR-222-3p	12.93	13.66	13.57	13.38	0.32	10.08398807
hsa-miR-574-3p	13.72	13.83	14.34	13.96	0.27	6.762989192
hsa-miR-191-5p	13.75	13.90	14.25	13.97	0.21	6.730254585
hsa-miR-1274B	14.28	14.08	13.84	14.06	0.18	6.295528960
hsa-miR-320a-3p	14.35	14.78	14.81	14.65	0.21	4.197880228
hsa-miR-484	14.82	14.88	14.95	14.89	0.05	3.563581189
hsa-miR-197-3p	15.65	16.11	15.73	15.83	0.20	1.854025114
hsa-miR-125b-5p	15.98	16.10	15.70	15.93	0.17	1.733067865
hsa-miR-99a-5p	15.98	15.97	16.34	16.09	0.17	1.542204224
hsa-miR-145-5p	15.98	15.95	16.49	16.14	0.25	1.494152546
hsa-miR-19b-3p	15.99	16.43	16.02	16.15	0.20	1.486233896
hsa-miR-214-3p	15.79	16.40	16.37	16.18	0.28	1.447598052
hsa-miR-21-5p	16.71	16.61	16.48	16.60	0.10	1.086479443
hsa-miR-342-3p	16.33	16.67	16.86	16.62	0.22	1.070779162
hsa-miR-132-3p	16.69	16.77	16.64	16.70	0.05	1.011849009
hsa-miR-16-5p	16.55	16.69	16.94	16.73	0.16	0.995615635
hsa-miR-523-3p	17.79	16.91	15.53	16.74	0.93	0.983952511
hsa-miR-409-3p	17.10	16.84	16.96	16.97	0.11	0.841476316
hsa-miR-221-3p	16.92	16.97	17.08	16.99	0.06	0.829124978
hsa-miR-1274A	17.21	16.95	16.94	17.03	0.12	0.803847975
hsa-miR-636	22.93	15.48	12.76	17.06	4.30	0.790403154
hsa-let-7b-5p	17.78	16.10	17.35	17.08	0.71	0.780602681
hsa-miR-210-3p	16.83	17.27	17.13	17.08	0.18	0.779341548
hsa-miR-29a-3p	16.82	17.47	17.27	17.19	0.27	0.722629345
hsa-miR-30b-5p	17.38	17.40	17.32	17.36	0.03	0.639490414
hsa-miR-106a-5p	17.23	17.58	17.47	17.43	0.15	0.610894287
hsa-miR-17-5p	17.39	17.49	17.44	17.44	0.04	0.606534357
hsa-miR-30c-5p	17.78	17.39	17.33	17.50	0.20	0.582633817
hsa-miR-720	17.94	17.54	17.49	17.66	0.20	0.521351303
hsa-miR-92a-3p	17.43	17.95	17.63	17.67	0.21	0.517749975
hsa-miR-483-5p	17.77	17.66	17.58	17.67	0.08	0.516555300
hsa-miR-20a-5p	18.22	18.29	17.88	18.13	0.18	0.376397024
hsa-miR-138-5p	17.69	18.53	18.18	18.13	0.34	0.375008235
hsa-miR-193a-5p	18.03	18.53	18.51	18.36	0.23	0.321448809
hsa-miR-382-5p	17.56	19.12	18.50	18.39	0.64	0.313599274
hsa-miR-28-3p	18.39	18.76	18.57	18.57	0.15	0.276431568
hsa-miR-31-5p	18.40	18.81	18.52	18.58	0.17	0.275793598
hsa-miR-199a-3p	18.20	18.97	18.89	18.69	0.35	0.255134201
hsa-miR-376c-3p	18.97	19.09	19.76	19.28	0.35	0.169888586
hsa-miR-520e-3p	20.11	22.56	15.28	19.32	3.03	0.165204860
hsa-miR-29c-3p	15.98	21.49	20.49	19.32	2.40	0.164899729
hsa-let-7e-5p	18.14	19.62	20.32	19.36	0.91	0.160575943
hsa-miR-152-3p	19.69	19.39	19.31	19.46	0.16	0.149097433
hsa-miR-30a-3p	19.88	18.79	19.82	19.50	0.50	0.145490183

**Table 4:** OA-related factors targeted by EV-miRNAs.

	EXPRESSING CELL (>1%)				% WEIGHT	MAIN EV-miRNA (%)	FUNCTION
	C	S	H	T			
**CYTOKINES**							
TNF		X	X		2.34	hsa-miR-125b-5p (1.73)	Pro-inflammatory
IL1B		X	X		1.09	hsa-miR-21-5p (1.09)	Pro-inflammatory
IL1A		X	X		6.73	hsa-miR-191-5p (6.73)	Pro-inflammatory
CSF1	X	X	X		0.15	hsa-miR-152-3p (0.15)	Macrophage activator
CXCL12		X	X		1.11	hsa-miR-221-3p (0.83)	Articular cartilage matrix degeneration
CCL5		X	X	X	1.45	hsa-miR-214-3p (1.45)	Cartilage erosion
IL11	X	X	X		0.58	hsa-miR-30c-5p (0.58)	Pro-inflammatory
**GROWTH FACTORS**							
TGFB1	X	X	X	X	26.88	hsa-miR-24-3p (20.12)	Chondrocytes homeostasis, hypertrophy
IGF1		X	X		1.14	hsa-miR-29a-3p (0.72)	Promote chondrocyte anabolic activity
FGF2	X	X			1.15	hsa-miR-16-5p (1.00)	Promote catabolic and anti-anabolic effects
BMP2	X	X	X		1.22	hsa-miR-106a-5p (0.61)	Promote cartilage regeneration
VEGFA	X	X	X		10.52	hsa-miR-320a-3p (4.20)	Chondrocyte catabolism
HGF		X	X		1.26	hsa-miR-16-5p (1.00)	Osteophyte formation
ANGPT2		X	X		3.22	hsa-miR-125b-5p (1.73)	Abnormal angiogenesis in OA
CTGF	X	X	X		2.07	hsa-miR-145-5p (1.49)	Osteophyte formation and ECM degradation
KITLG	X	X	X		4.20	hsa-miR-320a-3p (4.20)	Mast cell hyperplasia and inflammation
TGFB2	X	X	X		2.58	hsa-miR-145-5p (1.49)	High level in joint tissue during OA
IGF2	X	X			1.73	hsa-miR-125b-5p (1.73)	Promote cartilage matrix levels
BDNF		X			2.79	hsa-miR-132-3p (1.01)	Promote joint pain and inflammation
**PROTEASES**							
ADAM12	X	X			0.88	hsa-miR-29a-3p (0.72)	Proteinase involved in ECM degradation
ADAM17	X	X	X		1.64	hsa-miR-145-5p (1.49)	Proteinase involved in ECM degradation
ADAMTS9		X			0.72	hsa-miR-29a-3p (0.72)	Proteinase involved in ECM degradation
MMP1		X			11.27	hsa-miR-222-3p (10.08)	Proteinase involved in ECM degradation
MMP2	X	X			4.05	hsa-miR-125b-5p (1.73)	Proteinase involved in ECM degradation
MMP9		X	X		1.17	hsa-miR-132-3p (1.01)	Proteinase involved in ECM degradation
MMP14	X	X			21.61	hsa-miR-24-3p (20.12)	Proteinase involved in ECM degradation
PLAU		X	X		11.35	hsa-miR-193b-3p (11.35)	ECM-degrading enzyme
PLAT	X	X			1.09	hsa-miR-21-5p (1.09)	ECM-degrading enzyme
APC	X	X			2.34	hsa-miR-125b-5p (1.73)	Promote MMP activity
TIMP2	X	X			0.99	hsa-miR-106a-5p (0.61)	MMP inhibitor
TIMP3	X	X			12.61	hsa-miR-222-3p (10.08)	MMP inhibitor

**Table 5:** First quartile EV-miRNAs’ role in OA-related tissues and cells.

	% WEIGHT	ROLE
**CARTILAGE**		
Protective		
hsa-miR-24-3p	20.12	Prevents ECM degradation, increases chondrocyte viability
hsa-miR-193b-3p	11.35	Reduce cartilage degradation
hsa-miR-222-3p	10.08	Reduce cartilage degradation
hsa-miR-320a-3p	4.20	Increase chondrocyte viability
hsa-miR-125b-5p	1.73	Prevents aggrecan loss
hsa-miR-221-3p	0.83	Prevents ECM degradation
hsa-miR-210-3p	0.78	Promotes chondrocyte and ECM deposition
hsa-miR-17-5p	0.61	Induces autophagy
hsa-miR-92a-3p	0.52	Increases collagen deposition
hsa-miR-199a-3p	0.26	Anti-catabolic
hsa-miR-30a-3p	0.15	cartilage homeostasis
**TOTAL**	**50.62**	
Destructive		
hsa-miR-21-5p	1.09	Negatively regulates chondrogenesis
hsa-miR-16-5p	1.00	Cartilage degradation
hsa-miR-30b-5p	0.64	Pro-apoptotic, ECM degradation
hsa-miR-483-5p	0.52	Chondrocyte hypertrophy, ECM degradation and cartilage angiogenesis
hsa-miR-138-5p	0.38	Cartilage degradation
**TOTAL**	**3.61**	
*Dual*		
hsa-miR-145-5p	1.49	Regulates chondrocyte proliferation and fibrosis
**SYNOVIUM**		
Protective		
hsa-miR-29a-3p	0.72	Anti-fibrotic effects
**MACROPHAGE**		
*M1*		
hsa-miR-125b-5p	1.73	Pro-M1
hsa-miR-145-5p	1.49	Pro-M1
**TOTAL**	**3.23**	
*M2*		
hsa-miR-24-3p	20.12	Pro-M2, anti-M1
hsa-miR-222-3p	10.08	Pro-M2
hsa-let-7b-5p	0.78	Pro-M2
**TOTAL**	**30.99**	
**T CELL**		
*Pro-Activation*		
hsa-miR-19b-3p	1.49	Reduces PTEN repressor
hsa-miR-214-3p	1.45	Reduces PTEN repressor
hsa-miR-21-5p	1.09	Reduces PTEN repressor
hsa-miR-132-3p	1.01	Downregulate PIK3R1
hsa-miR-221-3p	0.83	Downregulate PIK3R1
hsa-let-7b-5p	0.78	Represses IL10
hsa-miR-106a-5p	0.61	Represses IL10
hsa-miR-17-5p	0.61	Reduces PTEN repressor and promotes IFNγ
hsa-let-7e-5p	0.16	Represses IL10
**TOTAL**	**8.02**	
*Anti-Activation*		
hsa-miR-24-3p	20.12	Represses IFNγ in activated CD4^+^ and CD8^+^
hsa-miR-125b-5p	1.73	Maintains T cell naïve state
hsa-miR-342-3p	1.07	Downregulated upon activation
**TOTAL**	**22.93**	
*Dual*		
hsa-miR-31-5p	0.28	Upregulates IL2, downregulated with activation
hsa-miR-210-3p	0.78	Upregulated in activated T cell, represses IL17
**TOTAL**	**1.06**	

## Data Availability

The raw data presented in this study are openly available in OSF at https://osf.io/ax8kg/?view_only=864bc5fca8924fea8f67a7fa3ba3e08a, created on 12 May 2022.

## Supplementary Materials

The following supporting information can be downloaded at: https://www.mdpi.com/article/10.3390/ijms232415908/s1, Table S1: PRP characteristics; Table S2: Soluble factors in activated-PRP; Table S3: Soluble factors in PRP-treated BMSCs: Table S4: Proteins defining the GO terms in Figure 3; Table S5: EV-miRNAs detected in PRP-treated BMSCs secretomes; Table S6: Experimentally validated targets for first quartile EV-miRNAs; Table S7: Univocal first quartile EV-miRNA targets.
