# Supplementary material for: Tissue-Protective and Anti-Inflammatory Landmark of PRP-Treated Mesenchymal Stromal Cells Secretome for Osteoarthritis
Source: Int J Mol Sci. 2022 Dec 14;23(24):15908. doi: 10.3390/ijms232415908 (PMC9788137; doi:10.3390/ijms232415908)
Supplement: Supplementary file 1 [file ijms-23-15908-s001.zip › Table S1.pdf]

Table S1. PRP characteristics

PATIENT	AGE yo	WBC (10^3 x μl)	RBC (10^6 x μl)	PLT (10^3 x μl)
1	56	0.05	0.01	326
2	60	0.02	0.02	205
3	52	0.01	0.03	235
4	56	0.03	0.01	314
5	62	0.01	0.01	292
6	74	0.05	0.03	360
7	62	0.01	0.02	239
8	50	0.03	0.00	336
9	50	0.02	0.00	276
10	38	0.00	0.00	419
11	32	0.02	0.00	365
12	37	0.07	0.01	293
13	68	0.00	0.01	287
14	32	0.02	0.01	334
15	74	0.08	0.02	375
16	71	0.01	0.01	216
17	85	0.01	0.02	471
18	59	0.01	0.02	206
19	74	0.00	0.01	284
20	74	0.06	0.01	259
21	41	0.08	0.01	383
22	55	0.15	0.01	340
23	49	0.06	0.01	319
24	48	0.02	0.02	391
25	52	0.04	0.01	297
26	59	0.04	0.02	408
27	82	0.12	0.05	392
28	16	0.50	0.07	549
29	47	1.64	0.04	485
MEAN	56	0.11	0.02	333
SD	16	0.30	0.02	83

WBC = white blood cells; RBC = red blood cells; PLT = platelets
