# Supplementary material for: Tissue-Protective and Anti-Inflammatory Landmark of PRP-Treated Mesenchymal Stromal Cells Secretome for Osteoarthritis
Source: Int J Mol Sci. 2022 Dec 14;23(24):15908. doi: 10.3390/ijms232415908 (PMC9788137; doi:10.3390/ijms232415908)
Supplement: Supplementary file 1 [file ijms-23-15908-s001.zip › Table S2.pdf]

Table S2. Soluble factors in pooled activated-PRP

TYPE	FACTOR	(pg/ml)	
CYT	ICAM2	790,983	Intercellular adhesion molecule 2
CYT	PLG	182,992	Plasminogen
REC	VCAM1	102,461	Vascular cell adhesion protein 1
CYT	IL13RA2	94,449	Interleukin-13 receptor subunit alpha-2
CHE	IL9	73,686	Interleukin-9
GF	IGFBP4	72,637	Insulin-like growth factor-binding protein 4
REC	MICB	66,893	MHC class I polypeptide-related sequence B
GF	TGFB1	58,819	Transforming growth factor beta-1
CHE	PF4	56,183	Platelet factor 4
CYT	IL23A	47,225	Interleukin-23 subunit alpha
CYT	FLT1	47,188	Vascular endothelial growth factor receptor 1
INF	PDGFB	47,171	Platelet-derived growth factor subunit B
CHE	IL31	38,293	Interleukin-31
GF	BMP7	35,835	Bone morphogenetic protein 7
CYT	SIGLEC5	35,491	Sialic acid-binding Ig-like lectin 5
GF	IGFBP3	34,608	Insulin-like growth factor-binding protein 3
CHE	CCL26	34,511	C-C motif chemokine 26
GF	FGF4	33,301	Fibroblast growth factor 4
CHE	SPP1	30,604	Osteopontin
GF	BMP4	28,461	Bone morphogenetic protein 4
GF	IGFBP2	27,669	Insulin-like growth factor-binding protein 2
REC	IL21R	26,521	Interleukin-21 receptor
REC	SELL	25,729	L-selectin
GF	CSF1R	24,163	Macrophage colony-stimulating factor 1 receptor
CYT	IL6ST	23,811	Interleukin-6 receptor subunit beta
INF	IL6R	23,412	Interleukin-6 receptor subunit alpha
CHE	CCL21	23,105	C-C motif chemokine 21
INF	TIMP2	22,795	Metalloproteinase inhibitor 2
REC	TNFRSF17	22,355	Tumor necrosis factor receptor superfamily member 17
CHE	IFNL1	19,506	Interferon lambda-1
CYT	CDH1	17,919	Cadherin-1
CYT	IL13RA1	16,766	Interleukin-13 receptor subunit alpha-1
GF	KDR	15,881	Vascular endothelial growth factor receptor 2
CYT	TREM1	15,662	Triggering receptor expressed on myeloid cells 1
CYT	CTSS	15,538	Cathepsin S
REC	TNFRSF18	13,410	Tumor necrosis factor receptor superfamily member 18
CYT	LGALS7	12,956	Galectin-7
CYT	TNFRSF10D	12,579	Tumor necrosis factor receptor superfamily member 10D
CYT	PDGFA	12,454	Platelet-derived growth factor subunit A
INF	TNFRSF1A	12,348	Tumor necrosis factor receptor superfamily member 1A
GF	TGFA	12,273	Protransforming growth factor alpha
INF	TIMP1	12,041	Metalloproteinase inhibitor 1
GF	FGF7	11,984	Fibroblast growth factor 7
CYT	IL2RB	11,830	Interleukin-2 receptor subunit beta
CYT	SERPINE1	11,194	Plasminogen activator inhibitor 1

CHE	CXCL16	10,931	C-X-C motif chemokine 16
INF	ICAM1	10,930	Intercellular adhesion molecule 1
INF	CCL5	10,751	C-C motif chemokine 5
INF	TNFRSF1B	10,501	Tumor necrosis factor receptor superfamily member 1B
GF	NTF3	10,424	Neurotrophin-3
CHE	IL17F	9,879	Interleukin-17F
CHE	CCL14	8,462	C-C motif chemokine 14
REC	PI3	8,259	Elafin
REC	PDGFRB	8,247	Platelet-derived growth factor receptor beta
CHE	CCL28	8,239	C-C motif chemokine 28
CHE	IL18BP	7,960	Interleukin-18-binding protein
CHE	CCL19	7,853	C-C motif chemokine 19
INF	IL16	7,502	Pro-interleukin-16
CYT	INHBA	7,178	Inhibin beta A chain
REC	SCARB2	7,022	Lysosome membrane protein 2
REC	ICAM3	6,991	Intercellular adhesion molecule 3
CYT	ANGPT1	6,757	Angiopoietin-1
GF	KIT	6,741	Mast/stem cell growth factor receptor Kit
REC	ALCAM	6,665	CD166 antigen
REC	PLAUR	6,647	Urokinase plasminogen activator surface receptor
REC	CNTN2	6,612	Contactin-2
REC	CD14	6,420	Monocyte differentiation antigen CD14
CYT	PARN	6,326	Poly(A)-specific ribonuclease PARN
GF	NGFR	6,284	Tumor necrosis factor receptor superfamily member 16
CHE	MST1	6,258	Hepatocyte growth factor-like protein
CYT	NRCAM	6,258	Neuronal cell adhesion molecule
GF	IGFBP1	6,101	Insulin-like growth factor-binding protein 1
REC	TYRO3	5,838	Tyrosine-protein kinase receptor TYRO3
CYT	TEK	5,792	Angiopoietin-1 receptor
INF	CXCL9	5,743	C-X-C motif chemokine 9
GF	NTF4	5,576	Neurotrophin-4
REC	TNFRSF9	5,347	Tumor necrosis factor receptor superfamily member 9
REC	IL10RB	5,287	Interleukin-10 receptor subunit beta
CHE	CCL27	5,141	C-C motif chemokine 27
REC	PECAM1	5,051	Platelet endothelial cell adhesion molecule
CYT	RETN	4,998	Resistin
REC	ERBB3	4,987	Receptor tyrosine-protein kinase erbB-3
CHE	MIF	4,895	Macrophage migration inhibitory factor
INF	LTA	4,534	Lymphotoxin-alpha
GF	EGFR	4,510	Epidermal growth factor receptor
CYT	IL1RL1	4,376	Interleukin-1 receptor-like 1
REC	LCN2	4,231	Neutrophil gelatinase-associated lipocalin
GF	IGFBP6	4,086	Insulin-like growth factor-binding protein 6
CYT	IL17B	4,012	Interleukin-17B
CHE	CXCL11	3,998	C-X-C motif chemokine 11
CHE	XCL1	3,946	Lymphotactin
GF	FIGF	3,883	Vascular endothelial growth factor D

REC	CD80	3,874	T-lymphocyte activation antigen CD80
CYT	CXCL12	3,873	C-X-C motif chemokine 12
GF	TGFB3	3,557	Transforming growth factor beta-3
REC	TNFRSF14	3,188	Tumor necrosis factor receptor superfamily member 14
GF	GDNF	3,063	Glial cell line-derived neurotrophic factor
GF	INS	3,020	Insulin
CYT	DKK1	2,983	Dickkopf-related protein 1
GF	VEGFA	2,961	Vascular endothelial growth factor A
REC	MOK	2,895	MAPK/MAK/MRK overlapping kinase
INF	CCL1	2,893	C-C motif chemokine 1
REC	HAVCR1	2,844	Hepatitis A virus cellular receptor 1
GF	HGF	2,830	Hepatocyte growth factor
REC	SELE	2,816	E-selectin
REC	TNFRSF10C	2,815	Tumor necrosis factor receptor superfamily member 10C
CYT	TGFB2	2,762	Transforming growth factor beta-2
GF	IGF1	2,745	Insulin-like growth factor I
CYT	THPO	2,721	Thrombopoietin
CYT	ANG	2,705	Angiogenin
GF	AREG	2,676	Amphiregulin
REC	TNFRSF21	2,674	Tumor necrosis factor receptor superfamily member 21
CYT	CED	2,572	Diaphyseal Dysplasia 1
INF	IL1A	2,482	Interleukin-1 alpha
INF	IL15	2,412	Interleukin-15
REC	MICA	2,364	MHC class I polypeptide-related sequence A
REC	ENG	2,181	Endoglin
CHE	TSLP	2,173	Thymic stromal lymphopoietin
INF	IL11	2,171	Interleukin-11
INF	CCL24	2,149	C-C motif chemokine 24
GF	PDGF1	2,145	Platelet-Derived Growth Factor A Chain
REC	CEACAM1	2,136	Carcinoembryonic antigen-related cell adhesion molecule 1
CHE	CXCL10	2,121	C-X-C motif chemokine 10
REC	LYVE1	2,111	Lymphatic vessel endothelial hyaluronic acid receptor 1
REC	IL17RA	2,081	Interleukin-17 receptor A
CYT	FCGR2B	2,076	Low affinity Ig Fc region receptor II-b
INF	CCL11	1,995	Eotaxin
GF	BMP5	1,847	Bone morphogenetic protein 5
CYT	FST	1,746	Follistatin
CHE	LIF	1,683	Leukemia inhibitory factor
CYT	IL2RA	1,581	Interleukin-2 receptor subunit alpha
CHE	AXL	1,571	Tyrosine-protein kinase receptor UFO
CHE	CCL22	1,561	C-C motif chemokine 22
CHE	TNFSF14	1,521	Tumor necrosis factor ligand superfamily member 14
REC	FAS	1,424	Tumor necrosis factor receptor superfamily member 6
GF	KITLG	1,366	Kit ligand
CHE	CXCL5	1,347	C-X-C motif chemokine 5
CYT	VEGFC	1,231	Vascular endothelial growth factor C
CHE	IFNL2	1,121	Interferon lambda-2

REC	NRG1	1,025	Pro-neuregulin-1, membrane-bound isoform
INF	CCL3	986	C-C motif chemokine 3
CHE	CCL16	949	C-C motif chemokine 16
INF	IL1B	932	Interleukin-1 beta
CHE	CCL23	865	Myeloid progenitor inhibitory factor
CYT	TDGF1	836	Teratocarcinoma-derived growth factor 1
CYT	EPCAM	806	Epithelial cell adhesion molecule
GF	HBEGF	793	Proheparin-binding EGF-like growth factor
INF	CSF3	780	Granulocyte colony-stimulating factor
CHE	CCL25	741	C-C motif chemokine 25
GF	FLT4	738	Vascular endothelial growth factor receptor 3
INF	IL1RN	729	Interleukin-1 receptor antagonist protein
REC	EDA2R	723	Tumor necrosis factor receptor superfamily member 27
CYT	CD40	703	Tumor necrosis factor receptor superfamily member 5
INF	CCL2	703	C-C motif chemokine 2
CYT	SHH	695	Sonic hedgehog protein
REC	TNFRSF8	678	Tumor necrosis factor receptor superfamily member 8
GF	GH1	657	Somatotropin
INF	IL4	528	Interleukin-4
GF	GDF15	519	Growth/differentiation factor 15
REC	CD40LG	477	CD40 ligand
GF	BDNF	464	Brain-derived neurotrophic factor
CYT	AGRP	455	Agouti-related protein
INF	CCL15	448	C-C motif chemokine 15
GF	PGF	426	Placenta growth factor
GF	NGF	383	Beta-nerve growth factor
INF	IL7	375	Interleukin-7
CHE	CCL8	333	C-C motif chemokine 8
GF	FGF2	317	Fibroblast growth factor 2
GF	PROK1	317	Prokineticin-1
REC	IL1R1	290	Interleukin-1 receptor type 1
INF	CXCL8	286	Interleukin-8
CHE	BTC	284	Probetacellulin
CHE	CCL17	274	C-C motif chemokine 17
CHE	SDF1	264	Stromal cell-derived factor 1
CHE	CCL18	240	C-C motif chemokine 18
INF	IL17A	225	Interleukin-17A
CHE	PPBP	224	Platelet basic protein
CHE	CCL7	185	C-C motif chemokine 7
INF	IL12B	179	Interleukin-12 subunit beta
REC	FLT3LG	168	Fms-related tyrosine kinase 3 ligand
CHE	CXCL6	165	C-X-C motif chemokine 6
CHE	CXCL1	145	Growth-regulated alpha protein
CHE	CCL20	144	C-C motif chemokine 20
INF	IL2	136	Interleukin-2
INF	CXCL13	132	C-X-C motif chemokine 13
INF	TNF	121	Tumor necrosis factor

REC	IL2RG	114	Cytokine receptor common subunit gamma
CYT	FASLG	105	Tumor necrosis factor ligand superfamily member 6
CHE	CCL13	99	C-C motif chemokine 13
INF	IL5	80	Interleukin-5
GF	TNFRSF11B	76	Tumor necrosis factor receptor superfamily member 11B
INF	CSF2	68	Granulocyte-macrophage colony-stimulating factor
INF	CCL4	55	C-C motif chemokine 4
INF	IFNG	54	Interferon gamma
GF	EGF	42	Pro-epidermal growth factor
INF	IL13	42	Interleukin-13
INF	CSF1	38	Macrophage colony-stimulating factor 1
INF	IL6	28	Interleukin-6
INF	IL10	18	Interleukin-10
INF	IL12A	14	Interleukin-12 subunit alpha

CHE = chemokine; CYT = cytokine; GF; growth factor; INF = inflammation; REC = receptor
