# Supplementary material for: Tissue-Protective and Anti-Inflammatory Landmark of PRP-Treated Mesenchymal Stromal Cells Secretome for Osteoarthritis
Source: Int J Mol Sci. 2022 Dec 14;23(24):15908. doi: 10.3390/ijms232415908 (PMC9788137; doi:10.3390/ijms232415908)
Supplement: Supplementary file 1 [file ijms-23-15908-s001.zip › Table S3.pdf]

Table S3. Soluble factors in PRP-treated BMSCs

		pg/milion cells					
TYPE	FACTOR	B1	B2	B3	MEAN	SD	
GF	IGFBP4	146,361	166,965	160,221	157,849	8,577	Insulin-like growth factor-binding protein 4
CHE	PF4	118,491	127,082	177,384	140,985	25,975	Platelet factor 4
GF	IGFBP3	67,919	65,875	69,956	67,917	1,666	Insulin-like growth factor-binding protein 3
GF	TGFB1	25,432	43,973	51,332	40,245	10,897	Transforming growth factor beta-1
INF	TIMP2	37,691	43,548	37,878	39,706	2,718	Metalloproteinase inhibitor 2
CHE	IFNL1	25,976	26,330	49,299	33,869	10,912	Interferon lambda-1
CYT	INHBA	25,821	24,754	27,428	26,001	1,099	Inhibin beta A chain
GF	VEGFA	18,261	24,211	24,511	22,328	2,878	Vascular endothelial growth factor A
INF	TIMP1	19,604	23,458	20,658	21,240	1,626	Metalloproteinase inhibitor 1
CYT	SERPINE1	20,131	20,479	16,545	19,052	1,778	Plasminogen activator inhibitor 1
GF	BMP4	6,102	33,281	8,863	16,082	12,213	Bone morphogenetic protein 4
GF	IGFBP6	9,679	14,168	13,933	12,593	2,063	Insulin-like growth factor-binding protein 6
GF	IGFBP2	5,173	7,631	10,814	7,873	2,309	Insulin-like growth factor-binding protein 2
CHE	XCL1	5,106	6,558	9,243	6,969	1,714	Lymphotactin
CHE	MIF	4,093	7,092	8,971	6,719	2,009	Macrophage migration inhibitory factor
CHE	MST1	3,693	8,731	5,406	5,943	2,092	Hepatocyte growth factor-like protein
INF	CCL5	5,185	6,404	5,895	5,828	500	C-C motif chemokine 5
CHE	CXCL16	4,085	5,879	6,742	5,569	1,107	C-X-C motif chemokine 16
CHE	CCL21	1,361	6,564	7,156	5,027	2,603	C-C motif chemokine 21
INF	TNFRSF1A	5,067	5,022	4,075	4,721	458	TNF receptor superfamily member 1A
CHE	CCL27	4,607	3,765	3,947	4,106	362	C-C motif chemokine 27
CHE	SPP1	2,646	3,255	5,516	3,806	1,235	Osteopontin
CHE	CXCL11	2,144	3,428	5,800	3,791	1,514	C-X-C motif chemokine 11
CYT	ICAM2	192	4,048	4,472	2,904	1,926	Intercellular adhesion molecule 2
CHE	CCL26	3,351	1,814	1,623	2,263	774	C-C motif chemokine 26
CYT	ANGPT1	2,234	2,211	2,087	2,177	65	Angiopoietin-1
CYT	IL6ST	1,590	1,619	3,224	2,144	764	Interleukin-6 receptor subunit beta
CHE	LIF	894	1,384	3,839	2,039	1,289	Leukemia inhibitory factor
CHE	CCL25	2,510	2,043	1,560	2,038	388	C-C motif chemokine 25
CYT	ANG	1,590	1,816	1,994	1,800	166	Angiogenin
CYT	CTSS	1,308	1,775	1,703	1,595	205	Cathepsin S
CYT	DKK1	1,182	2,074	1,385	1,547	382	Dickkopf-related protein 1
GF	HGF	632	1,850	1,899	1,460	586	Hepatocyte growth factor
CHE	TNFSF14	454	1,423	2,160	1,346	698	TNF ligand superfamily member 14
CYT	IL23A	645	1,270	1,467	1,127	350	Interleukin-23 subunit alpha
GF	KDR	607	1,327	1,426	1,120	365	Vascular endothelial growth factor receptor 2
REC	PLAUR	1,300	879	1,158	1,112	175	Urokinase plasminogen activator surface receptor
REC	ALCAM	996	1,004	1,219	1,073	104	CD166 antigen
GF	FGF7	235	1,518	1,447	1,066	589	Fibroblast growth factor 7
CHE	IFNL2	1,048	851	1,121	1,006	114	Interferon lambda-2
INF	CCL2	1,193	744	753	897	209	C-C motif chemokine 2
CHE	CXCL10	664	725	1,080	823	183	C-X-C motif chemokine 10
CHE	BTC	387	995	717	700	249	Probetacellulin
CYT	CDH1	169	108	1,786	687	777	Cadherin-1

CYT	FST	592	778	665	679	77	Follistatin
CHE	AXL	443	577	991	670	233	Tyrosine-protein kinase receptor UFO
GF	GDF15	466	729	721	639	122	Growth/differentiation factor 15
CHE	CCL28	955	25	864	615	419	C-C motif chemokine 28
CYT	CED	747	468	621	612	114	Diaphyseal Dysplasia 1
INF	CXCL8	520	720	424	555	123	Interleukin-8
INF	IL6	625	578	382	528	105	Interleukin-6
INF	IL1RN	502	545	481	509	27	Interleukin-1 receptor antagonist protein
GF	IGFBP1	150	442	763	452	250	Insulin-like growth factor-binding protein 1
CYT	IL17B	101	533	630	421	230	Interleukin-17B
INF	IL6R	328	361	456	382	54	Interleukin-6 receptor subunit alpha
CYT	IL2RB	193	655	275	374	202	Interleukin-2 receptor subunit beta
GF	NTF4	140	452	456	349	148	Neurotrophin-4
GF	AREG	38	356	604	333	231	Amphiregulin
GF	NTF3	24	314	561	300	219	Neurotrophin-3
CHE	CXCL12	274	254	302	277	20	C-X-C motif chemokine 12
INF	IL1A	231	380	208	273	76	Interleukin-1 alpha
INF	TNFRSF1B	180	297	299	259	56	TNF receptor superfamily member 1B
GF	EGFR	167	268	268	234	48	Epidermal growth factor receptor
GF	FIGF	193	262	220	225	28	Vascular endothelial growth factor D
REC	SCARB2	239	209	193	214	19	Lysosome membrane protein 2
INF	ICAM1	180	177	219	192	19	Intercellular adhesion molecule 1
REC	CD14	243	112	196	184	54	Monocyte differentiation antigen CD14
CHE	CCL8	126	105	196	142	39	C-C motif chemokine 8
CHE	CCL7	138	122	164	141	17	C-C motif chemokine 7
INF	IL16	147	236	28	137	85	Pro-interleukin-16
INF	CXCL9	82	174	154	137	40	C-X-C motif chemokine 9
CHE	CCL20	106	152	135	131	19	C-C motif chemokine 20
CYT	VEGFC	80	183	108	124	44	Vascular endothelial growth factor C
INF	CCL1	93	92	161	115	33	C-C motif chemokine 1
INF	CSF2	213	45	79	112	73	GM colony-stimulating factor
GF	TNFRSF11B	100	127	84	104	18	TNF receptor superfamily member 11B
INF	IL15	82	132	92	102	21	Interleukin-15
GF	PGF	51	122	127	100	35	Placenta growth factor
CYT	SDF1	43	79	143	88	42	Stromal cell-derived factor 1
CHE	PPBP	82	91	76	83	6	Platelet basic protein
REC	FAS	89	74	76	80	7	TNF receptor superfamily member 6
CYT	SIGLEC5	36	37	142	72	50	Sialic acid-binding Ig-like lectin 5
GF	PROK1	57	78	80	72	10	Prokineticin-1
INF	IL2	35	77	99	70	27	Interleukin-2
CYT	LGALS7	35	139	35	70	49	Galectin-7
GF	FLT4	24	107	49	60	34	Vascular endothelial growth factor receptor 3
INF	IFNG	54	23	99	59	31	Interferon gamma
CHE	CCL18	41	52	81	58	17	C-C motif chemokine 18
INF	IL1B	63	60	45	56	8	Interleukin-1 beta
CYT	CD40	32	17	115	55	43	TNF receptor superfamily member 5
CHE	CCL17	7	50	75	44	28	C-C motif chemokine 17

CYT	EPCAM	47	24	48	40	11	Epithelial cell adhesion molecule
INF	CSF3	56	24	32	38	14	Granulocyte colony-stimulating factor
CHE	CCL13	8	29	67	34	24	C-C motif chemokine 13
CYT	IL2RA	30	59	11	34	20	Interleukin-2 receptor subunit alpha
GF	GH1	33	25	42	34	7	Somatotropin
CHE	CCL14	5	22	72	33	29	C-C motif chemokine 14
REC	ENG	38	20	12	23	11	Endoglin
INF	CSF1	32	22	12	22	8	Macrophage colony-stimulating factor 1
REC	TNFRSF21	32	13	16	20	8	TNF receptor superfamily member 21
GF	BDNF	13	21	18	17	3	Brain-derived neurotrophic factor
INF	CCL4	5	6	4	5	1	C-C motif chemokine 4
CHE	CCL16	2	5	6	4	2	C-C motif chemokine 16
INF	IL12A	2	4	2	3	1	Interleukin-12 subunit alpha
INF	CXCL13	3	2	2	2	0	C-X-C motif chemokine 13

CHE = chemokine; CYT = cytokine; GF; growth factor; INF = inflammation; REC = receptor
