# Supplementary material for: Tissue-Protective and Anti-Inflammatory Landmark of PRP-Treated Mesenchymal Stromal Cells Secretome for Osteoarthritis
Source: Int J Mol Sci. 2022 Dec 14;23(24):15908. doi: 10.3390/ijms232415908 (PMC9788137; doi:10.3390/ijms232415908)
Supplement: Supplementary file 1 [file ijms-23-15908-s001.zip › Table S4.pdf]

Table S4 – Proteins defining the GO terms in Figure 3

- GO:0040011 Locomotion 54 PROTEINS
MIF,CCL17,TGFB1,HGF,CCL2,CCL1,CCL13,BMP4,CCL21,CCL27,IL1B,EPCAM,KDR,ICAM1,FGF7,EGFR,CXCL13,CXCL16,PPBP,PF4,FIGF,AXL,IGFBP6,IL16,IL12A,CXCL10,ALCAM,CXCL8,CXCL11,ANG,PLAUR,CCL20,CCL28,CXCL9,XCL1,IL6R,ENG,CCL7,CCL25,CCL8,CCL26,CXCL12,IL6,NTF3,BDNF,ANGPT1,PGF,CCL5,CCL16,VEGFA,CCL14,CCL18,VEGFC,CCL4

- GO:0006935 Chemotaxis 45 PROTEINS
MIF,CCL17,HGF,CCL2,CCL1,CCL13,BMP4,CCL21,CCL27,IL1B,FGF7,CXCL13,CXCL16,PPBP,PF4,FIGF,IL16,CXCL10,ALCAM,CXCL8,CXCL11,PLAUR,CCL20,CCL28,CXCL9,XCL1,IL6R,ENG,CCL7,CCL25,CCL8,CCL26,CXCL12,IL6,NTF3,BDNF,ANGPT1,PGF,CCL5,CCL16,VEGFA,CCL14,CCL18,VEGFC,CCL4

- GO:0030595 Leukocyte chemotaxis 30 PROTEINS
CCL17,CCL2,CCL1,CCL13,CCL21,IL1B,CXCL13,CXCL16,PPBP,PF4,IL16,CXCL10,CXCL8,CXCL11,CCL20,CXCL9,XCL1,IL6R,CCL7,CCL25,CCL8,CCL26,CXCL12,IL6,CCL5,CCL16,VEGFA,CCL14,CCL18,CCL4

- GO:0071621 Granulocyte chemotaxis 25 PROTEINS
CCL17,CCL2,CCL1,CCL13,CCL21,IL1B,CXCL13,PPBP,PF4,CXCL10,CXCL8,CXCL11,CCL20,CXCL9,XCL1,CCL7,CCL25,CCL8,CCL26,CCL5,CCL16,VEGFA,CCL14,CCL18,CCL4

- GO:0048247 Lymphocyte chemotaxis 21 PROTEINS
CCL17,CCL2,CCL1,CCL13,CCL21,CXCL13,CXCL16,CXCL10,CXCL11,CCL20,XCL1,CCL7,CCL25,CCL8,CCL26,CXCL12,CCL5,CCL16,CCL14,CCL18,CCL4

- GO:0002548 Monocyte chemotaxis 18 PROTEINS
CCL17,CCL2,CCL1,CCL13,CCL21,CCL20,XCL1,IL6R,CCL7,CCL25,CCL8,CCL26,IL6,CCL5,CCL16,CCL14,CCL18,CCL4

- GO:0030198 Extracellular matrix organization 12 PROTEINS
TIMP1,TGFB1,SERPINE1,CDH1,TIMP2,KDR,ICAM1,TNFRSF11B,CTSS,ENG,SPP1,ICAM2
