# Supplementary material for: Tissue-Protective and Anti-Inflammatory Landmark of PRP-Treated Mesenchymal Stromal Cells Secretome for Osteoarthritis
Source: Int J Mol Sci. 2022 Dec 14;23(24):15908. doi: 10.3390/ijms232415908 (PMC9788137; doi:10.3390/ijms232415908)
Supplement: Supplementary file 1 [file ijms-23-15908-s001.zip › Table S5.pdf]

Table S5 - EV-miRNAs detected in PRP-treated BMSCs secretomes

	CRT					
miRBase ID	B1	B2	B3	Mean	SD	Weight %
**hsa-miR-24-3p**	**12.24**	**12.53**	**12.40**	**12.39**	**0.12**	**20.12142652**
**hsa-miR-193b-3p**	**12.96**	**13.40**	**13.28**	**13.21**	**0.18**	**11.35032031**
**hsa-miR-222-3p**	**12.93**	**13.66**	**13.57**	**13.38**	**0.32**	**10.08398807**
**hsa-miR-574-3p**	**13.72**	**13.83**	**14.34**	**13.96**	**0.27**	**6.762989192**
**hsa-miR-191-5p**	**13.75**	**13.90**	**14.25**	**13.97**	**0.21**	**6.730254585**
**hsa-miR-1274B**	**14.28**	**14.08**	**13.84**	**14.06**	**0.18**	**6.295528960**
**hsa-miR-320a-3p**	**14.35**	**14.78**	**14.81**	**14.65**	**0.21**	**4.197880228**
**hsa-miR-484**	**14.82**	**14.88**	**14.95**	**14.89**	**0.05**	**3.563581189**
**hsa-miR-197-3p**	**15.65**	**16.11**	**15.73**	**15.83**	**0.20**	**1.854025114**
**hsa-miR-125b-5p**	**15.98**	**16.10**	**15.70**	**15.93**	**0.17**	**1.733067865**
**hsa-miR-99a-5p**	**15.98**	**15.97**	**16.34**	**16.09**	**0.17**	**1.542204224**
**hsa-miR-145-5p**	**15.98**	**15.95**	**16.49**	**16.14**	**0.25**	**1.494152546**
**hsa-miR-19b-3p**	**15.99**	**16.43**	**16.02**	**16.15**	**0.20**	**1.486233896**
**hsa-miR-214-3p**	**15.79**	**16.40**	**16.37**	**16.18**	**0.28**	**1.447598052**
**hsa-miR-21-5p**	**16.71**	**16.61**	**16.48**	**16.60**	**0.10**	**1.086479443**
**hsa-miR-342-3p**	**16.33**	**16.67**	**16.86**	**16.62**	**0.22**	**1.070779162**
**hsa-miR-132-3p**	**16.69**	**16.77**	**16.64**	**16.70**	**0.05**	**1.011849009**
**hsa-miR-16-5p**	**16.55**	**16.69**	**16.94**	**16.73**	**0.16**	**0.995615635**
**hsa-miR-523-3p**	**17.79**	**16.91**	**15.53**	**16.74**	**0.93**	**0.983952511**
**hsa-miR-409-3p**	**17.10**	**16.84**	**16.96**	**16.97**	**0.11**	**0.841476316**
**hsa-miR-221-3p**	**16.92**	**16.97**	**17.08**	**16.99**	**0.06**	**0.829124978**
**hsa-miR-1274A**	**17.21**	**16.95**	**16.94**	**17.03**	**0.12**	**0.803847975**
**hsa-miR-636**	**22.93**	**15.48**	**12.76**	**17.06**	**4.30**	**0.790403154**
**hsa-let-7b-5p**	**17.78**	**16.10**	**17.35**	**17.08**	**0.71**	**0.780602681**
**hsa-miR-210-3p**	**16.83**	**17.27**	**17.13**	**17.08**	**0.18**	**0.779341548**
**hsa-miR-29a-3p**	**16.82**	**17.47**	**17.27**	**17.19**	**0.27**	**0.722629345**
**hsa-miR-30b-5p**	**17.38**	**17.40**	**17.32**	**17.36**	**0.03**	**0.639490414**
**hsa-miR-106a-5p**	**17.23**	**17.58**	**17.47**	**17.43**	**0.15**	**0.610894287**
**hsa-miR-17-5p**	**17.39**	**17.49**	**17.44**	**17.44**	**0.04**	**0.606534357**
**hsa-miR-30c-5p**	**17.78**	**17.39**	**17.33**	**17.50**	**0.20**	**0.582633817**
**hsa-miR-720**	**17.94**	**17.54**	**17.49**	**17.66**	**0.20**	**0.521351303**
**hsa-miR-92a-3p**	**17.43**	**17.95**	**17.63**	**17.67**	**0.21**	**0.517749975**
**hsa-miR-483-5**	**17.77**	**17.66**	**17.58**	**17.67**	**0.08**	**0.51655530**
**hsa-miR-20a-5p**	**18.22**	**18.29**	**17.88**	**18.13**	**0.18**	**0.376397024**
**hsa-miR-138-5p**	**17.69**	**18.53**	**18.18**	**18.13**	**0.34**	**0.375008235**
**hsa-miR-193a-5p**	**18.03**	**18.53**	**18.51**	**18.36**	**0.23**	**0.321448809**
**hsa-miR-382-5p**	**17.56**	**19.12**	**18.50**	**18.39**	**0.64**	**0.313599274**
**hsa-miR-28-3p**	**18.39**	**18.76**	**18.57**	**18.57**	**0.15**	**0.276431568**
**hsa-miR-31-5p**	**18.40**	**18.81**	**18.52**	**18.58**	**0.17**	**0.275793598**
**hsa-miR-199a-3p**	**18.20**	**18.97**	**18.89**	**18.69**	**0.35**	**0.255134201**
**hsa-miR-376c-3p**	**18.97**	**19.09**	**19.76**	**19.28**	**0.35**	**0.169888586**
**hsa-miR-520e-3p**	**20.11**	**22.56**	**15.28**	**19.32**	**3.03**	**0.165204860**
**hsa-miR-29c-3p**	**15.98**	**21.49**	**20.49**	**19.32**	**2.40**	**0.164899729**

**hsa-let-7e-5p**	**18.14**	**19.62**	**20.32**	**19.36**	**0.91**	**0.160575943**
**hsa-miR-152-3p**	**19.69**	**19.39**	**19.31**	**19.46**	**0.16**	**0.149097433**
**hsa-miR-30a-3p**	**19.88**	**18.79**	**19.82**	**19.50**	**0.50**	**0.145490183**
hsa-miR-34a-5p	19.85	19.65	19.30	19.60	0.23	0.135559150
hsa-miR-186-5p	19.68	19.38	19.98	19.68	0.24	0.128721664
hsa-miR-212-3p	19.64	19.70	19.76	19.70	0.05	0.126510393
hsa-miR-99b-5p	19.97	19.93	19.50	19.80	0.21	0.118202154
hsa-miR-551b-3p	21.60	19.08	18.82	19.83	1.26	0.115529028
hsa-miR-376a-3p	19.91	19.94	19.90	19.92	0.02	0.108868710
hsa-miR-328-3p	19.92	20.15	19.78	19.95	0.15	0.106406704
hsa-miR-331-3p	19.82	20.20	20.07	20.03	0.16	0.100736689
hsa-miR-194-5p	19.33	21.03	19.98	20.11	0.70	0.095148708
hsa-miR-146a-5p	19.91	20.42	20.47	20.26	0.25	0.085713169
hsa-miR-149-5p	20.04	20.24	20.58	20.29	0.22	0.084396533
hsa-miR-425-5p	19.76	20.51	20.82	20.36	0.45	0.079899392
hsa-miR-143-3p	20.17	20.60	20.35	20.37	0.18	0.079402497
hsa-miR-130a-3p	20.48	20.72	20.16	20.45	0.23	0.075084592
hsa-miR-663b	21.03	20.36	20.59	20.66	0.28	0.064973455
hsa-miR-886-5p	20.52	20.74	20.79	20.68	0.12	0.064004982
hsa-miR-106b-5p	20.94	20.66	20.47	20.69	0.19	0.063768767
hsa-miR-335-5p	20.97	20.77	20.54	20.76	0.18	0.060678436
hsa-miR-26a-5p	20.90	21.02	20.51	20.81	0.21	0.058679249
hsa-miR-146b-5p	20.51	20.70	21.45	20.89	0.40	0.055680932
hsa-miR-365a-3p	20.89	21.02	20.79	20.90	0.10	0.055219720
hsa-miR-127-3p	21.55	20.69	20.70	20.98	0.40	0.052265197
hsa-miR-339-5p	20.99	20.69	21.37	21.02	0.28	0.050871267
hsa-miR-224-5p	21.43	20.41	21.29	21.04	0.45	0.049847366
hsa-miR-218-5p	20.99	21.09	21.12	21.07	0.05	0.049104337
hsa-miR-125a-5p	20.46	20.91	21.87	21.08	0.59	0.048720096
hsa-let-7a-5p	21.22	20.72	21.34	21.09	0.27	0.048238461
hsa-miR-31-3p	21.55	21.32	20.90	21.26	0.27	0.043035207
hsa-miR-590-5p	20.91	21.51	21.46	21.29	0.27	0.041974626
hsa-miR-155-5p	20.61	21.31	22.07	21.33	0.59	0.040921243
hsa-miR-134-5p	20.90	21.43	21.92	21.42	0.42	0.038517576
hsa-miR-532-5p	21.23	21.59	21.53	21.45	0.16	0.037663930
hsa-miR-345-5p	20.97	22.15	21.27	21.46	0.50	0.037282983
hsa-miR-301a-3p	21.70	21.85	21.06	21.54	0.35	0.035484355
hsa-let-7g-5p	21.60	21.14	21.89	21.54	0.31	0.035312633
hsa-miR-660-5p	21.39	21.68	21.86	21.64	0.19	0.032925011
hsa-miR-130b-3p	21.49	21.66	21.80	21.65	0.13	0.032826261
hsa-miR-10b-3p	21.90	21.54	21.57	21.67	0.16	0.032351888
hsa-miR-30e-3p	20.25	20.56	24.23	21.68	1.80	0.032069114
hsa-miR-7-1-3p	21.90	21.57	21.82	21.76	0.14	0.030325196
hsa-miR-27a-3p	21.46	21.99	21.87	21.77	0.23	0.030115733
hsa-miR-27b-3p	21.64	22.36	21.39	21.80	0.41	0.029536868
hsa-miR-423-5p	21.83	21.72	21.98	21.84	0.11	0.028722554
hsa-miR-339-3p	21.93	22.11	21.47	21.84	0.27	0.028722554

hsa-miR-1260a	21.92	20.86	22.85	21.88	0.81	0.028021172
hsa-miR-93-5p	21.42	22.20	22.10	21.90	0.35	0.027482579
hsa-miR-140-5p	21.96	21.99	21.91	21.95	0.03	0.026589381
hsa-miR-19a-3p	22.01	22.22	21.64	21.95	0.24	0.026546412
hsa-miR-494-3p	21.95	22.18	21.95	22.03	0.11	0.025254101
hsa-miR-193b-5p	21.91	22.10	22.20	22.07	0.12	0.024495516
hsa-miR-1290	22.19	21.67	22.55	22.14	0.36	0.023383923
hsa-miR-15b-5p	22.39	22.44	21.69	22.17	0.34	0.022812905
hsa-miR-23a-3p	22.49	22.18	22.15	22.27	0.15	0.021334434
hsa-miR-370-3p	22.29	22.65	22.03	22.33	0.25	0.020522172
hsa-miR-374a-5p	22.34	22.88	21.80	22.34	0.44	0.020295839
hsa-miR-99b-3p	21.94	22.44	22.65	22.35	0.30	0.020239649
hsa-miR-26b-5p	22.83	22.05	22.51	22.46	0.32	0.018671665
hsa-miR-30a-5p	23.16	22.25	22.11	22.51	0.46	0.018102434
hsa-miR-25-3p	22.74	22.36	22.44	22.51	0.16	0.018018982
hsa-miR-625-3p	22.30	22.17	23.19	22.55	0.46	0.017510060
hsa-let-7c-5p	21.92	22.52	23.59	22.68	0.69	0.016064213
hsa-miR-361-5p	22.24	22.77	23.09	22.70	0.35	0.015821111
hsa-miR-410-3p	22.94	22.64	22.58	22.72	0.16	0.015606896
hsa-miR-148a-3p	22.75	22.71	22.87	22.78	0.07	0.015019646
hsa-miR-10a-5p	22.55	22.88	22.91	22.78	0.16	0.014978057
hsa-miR-195-5p	22.76	23.16	22.73	22.88	0.20	0.013942771
hsa-miR-323a-3p	22.68	22.79	23.24	22.90	0.24	0.013760348
hsa-miR-432-3p	23.07	22.47	23.25	22.93	0.33	0.013514585
hsa-miR-708-5p	23.29	23.27	22.24	22.93	0.49	0.013458497
hsa-miR-34a-3p	23.91	22.31	22.66	22.96	0.69	0.013205911
hsa-miR-766-3p	23.80	22.83	22.37	23.00	0.60	0.012841826
hsa-miR-664a-3p	23.20	23.06	23.21	23.16	0.07	0.011523011
hsa-miR-532-3p	23.10	23.32	23.30	23.24	0.10	0.010896394
hsa-miR-34b-3p	24.08	23.10	22.83	23.34	0.54	0.010173739
hsa-miR-296-5p	23.87	23.63	22.64	23.38	0.53	0.009872702
hsa-miR-192-5p	22.23	24.11	23.85	23.39	0.83	0.009779623
hsa-miR-93-3p	23.44	23.20	23.57	23.40	0.15	0.009739036
hsa-miR-181a-5p	23.31	23.67	23.31	23.43	0.17	0.009551838
hsa-miR-324-3p	23.77	23.60	23.13	23.50	0.27	0.009109962
hsa-miR-744-5p	24.10	23.08	23.75	23.65	0.43	0.008214156
hsa-miR-28-5p	23.73	23.55	23.78	23.68	0.10	0.008000623
hsa-miR-454-3p	24.55	23.26	23.85	23.89	0.53	0.006955293
hsa-miR-223-3p	24.19	24.82	22.97	23.99	0.77	0.006452146
hsa-miR-103a-3p	24.03	23.81	24.19	24.01	0.16	0.006392786
hsa-miR-487b-3p	24.05	23.63	24.38	24.02	0.31	0.006338373
hsa-miR-539-5p	23.97	24.26	24.10	24.11	0.12	0.005952290
hsa-miR-374b-5p	23.66	24.70	24.27	24.21	0.42	0.005552401
hsa-miR-411-5p	23.99	24.34	24.50	24.28	0.21	0.005310247
hsa-miR-330-3p	24.99	24.04	23.90	24.31	0.48	0.005185364
hsa-miR-215-5p	22.99	24.38	25.89	24.42	1.18	0.004793608
hsa-miR-452-5p	23.93	24.79	24.87	24.53	0.42	0.004443752

hsa-miR-485-3p	24.40	24.63	24.58	24.53	0.10	0.004442726
hsa-miR-615-3p	24.72	24.79	24.45	24.65	0.15	0.004091922
hsa-miR-629-3p	25.91	24.00	24.23	24.72	0.85	0.003908944
hsa-miR-203a-3p	24.88	25.86	23.61	24.78	0.92	0.003731558
hsa-miR-886-3p	23.98	24.90	25.53	24.80	0.63	0.003682737
hsa-miR-433-3p	24.20	24.86	25.44	24.83	0.51	0.003610282
hsa-miR-185-5p	23.96	25.27	25.27	24.83	0.62	0.003606949
hsa-miR-493-3p	24.50	24.71	25.48	24.90	0.42	0.003446453
hsa-miR-491-5p	24.86	25.13	24.91	24.97	0.12	0.003292337
hsa-miR-425-3p	24.46	24.81	26.34	25.20	0.81	0.002792283
hsa-miR-140-3p	24.84	25.58	25.18	25.20	0.30	0.002791639
hsa-miR-1271-5p	25.51	26.33	24.09	25.31	0.93	0.002587895
hsa-miR-942-5p	24.73	25.42	26.22	25.45	0.61	0.002345310
hsa-miR-615-5p	25.12	26.85	25.04	25.67	0.83	0.002020593
hsa-miR-483-3p	25.38	23.91	28.28	25.86	1.82	0.001772901
hsa-miR-214-5p	25.35	26.84	25.62	25.94	0.65	0.001679205
hsa-miR-335-3p	25.84	25.25	26.81	25.96	0.64	0.001647691
hsa-miR-187-3p	26.34	26.27	25.71	26.11	0.28	0.001493584
hsa-miR-424-3p	25.93	26.41	26.13	26.16	0.20	0.001441707
hsa-miR-628-3p	25.84	25.88	26.86	26.19	0.47	0.001405203
hsa-miR-589-3p	25.86	25.91	27.00	26.26	0.52	0.001345161
hsa-miR-885-5p	26.09	27.72	25.05	26.28	1.10	0.001319915
hsa-miR-455-5p	26.55	26.89	25.50	26.31	0.59	0.001295144
hsa-miR-27b-5p	26.39	25.83	26.98	26.40	0.47	0.001220477
hsa-miR-133a-3p	25.36	26.55	27.56	26.49	0.90	0.001142963
hsa-miR-126-3p	25.77	26.91	26.81	26.50	0.52	0.001140325
hsa-miR-30d-3p	26.87	27.05	25.61	26.51	0.64	0.001127748
hsa-miR-10b-5p	26.75	27.12	25.70	26.52	0.61	0.001117632
hsa-miR-889-3p	28.98	25.32	25.44	26.58	1.70	0.001073840
hsa-miR-20b-5p	27.00	25.83	27.06	26.63	0.57	0.001037980
hsa-miR-22-5p	26.19	25.86	28.36	26.80	1.11	0.000921749
hsa-miR-24-2-5p	28.09	26.94	26.52	27.19	0.66	0.000706508
hsa-miR-1285-3p	27.83	27.27	26.65	27.25	0.48	0.000676477
hsa-miR-769-5p	28.64	27.13	26.23	27.33	0.99	0.000637330
hsa-miR-136-3p	28.21	25.74	28.41	27.45	1.22	0.000587412
hsa-miR-193a-3p	26.51	27.48	28.41	27.47	0.78	0.000580665
hsa-miR-500a-5p	27.53	28.88	27.02	27.81	0.79	0.000458008
hsa-miR-1291	28.21	27.50	27.74	27.82	0.30	0.000455581
hsa-miR-650	29.01	27.84	26.90	27.91	0.87	0.000426252
hsa-miR-18a-5p	28.78	27.84	27.27	27.96	0.62	0.000411637
hsa-miR-151a-5p	29.04	27.21	28.27	28.17	0.75	0.000356122
hsa-miR-130b-5p	27.75	28.40	28.48	28.21	0.33	0.000346785
hsa-miR-1255b-5p	29.90	28.17	29.12	29.06	0.71	0.000192258
hsa-miR-145-3p	31.60	28.41	29.31	29.77	1.34	0.000117612
hsa-miR-502-3p	31.28	29.53	28.99	29.93	0.98	0.000105339
hsa-miR-505-5p	29.68	30.79	29.92	30.13	0.48	9.19788E-05
hsa-miR-125a-3p	31.50	28.02	31.63	30.38	1.67	7.69702E-05

In **bold**, miRNAs falling in the first quartile
