# Supplementary material for: Tissue-Protective and Anti-Inflammatory Landmark of PRP-Treated Mesenchymal Stromal Cells Secretome for Osteoarthritis
Source: Int J Mol Sci. 2022 Dec 14;23(24):15908. doi: 10.3390/ijms232415908 (PMC9788137; doi:10.3390/ijms232415908)
Supplement: Supplementary file 1 [file ijms-23-15908-s001.zip › Table S6.pdf]

Table S6 - Experimentally validated targets for first quartile EV-miRNAs

miRNA	hsa-miR-24-3p	hsa-miR-193b-3p	hsa-miR-222-3p	hsa-miR-574-3p	hsa-miR-191-5p
Weight %	20.12	11.35	10.08	6.76	6.73
Targets	ABCB9	AKR1C2	ABCG2	CLTC	BASP1
	ACVR1B	CCND1	ADAM1A	CUL2	CCND2
	AGPAT2	ESR1	ARID1A	EGFR	CDK6
	ARHGAP19	ETS1	BBC3	EP300	CDK9
	ATG4A	KIT	BMF	RAC1	CEBPB
	AURKB	KRAS	CDKN1B	RXRA	CTDSP2
	BCAR1	MAX	CDKN1C	SMAD4	EGR1
	BCL2L11	MCL1	CERS2	TGFB1	IL1A
	BRCA1	MYB	CORO1A		LRRC8A
	CARD10	NF1	DICER1		MDM4
	CCNA2	PLAU	DIRAS3		NDST1
	CCND1	PRAP1	DKK2		NOTCH2
	CDK1	RAD51	ESR1		RPS6KA3
	CDK4	SHMT2	ETS1		SATB1
	CDKN1B	SMAD3	FOS		SLC16A2
	CDKN2A	YWHAZ	FOXO1		SOX4
	CHEK1		FOXO3		TMC7
	COPS5		GAS5		YBX3
	CORO1A		GJA1		
	CYP11B2		GNAI2		
	DEDD		GNAI3		
	DHFR		GRB10		
	DHFRP1		ICAM1		
	DND1		KIT		
	DYRK2		MGMT		
	E2F2		MMP1		
	EIF2S3		PLXNC1		
	FAF1		PPP2R2A		
	FBXW7		PRDM1		
	FEN1		PTEN		
	FGF11		RECK		
	FGFR3		RUNX2		
	FSCN1		SELE		
	FURIN		SMAD5		
	GATA3		SOD2		
	H2AFX		SSSCA1		
	HMOX1		SSX2IP		
	HNF4A		STAT5A		
	IFNG		TCEAL1		
	IFNR		TIMP3		
	IL4		TMED7		
	INSIG1		TNFSF10		
	JPH2		TP53		
	LDHA		TRPS1		
	LDHB		VGLL4		
	MAFB				

MAP3K9
MAPK14
MAPK7
MEN1
MLEC
MMP14
MT1M
MXI1
MYC
NCAN
NCSTN
NDST1
NOS3
NOTCH1
PAK4
PCNA
PDGFRB
POLD1
PRDX6
PRKCH
PSAP
PTPN9
PTPRF
REG4
S100A8
SH3PXD2A
SLC6A4
SP1
SSSCA1
ST7L
STX16
TACC3
TGFB1
TMED7
TMEM92
TNK2
TP53
TRIB3
TRIM11
WNT4
XIAP
ZNF217

hsa-miR-320a-3p	hsa-miR-484	hsa-miR-197-3p	hsa-miR-125b-5p	hsa-miR-99a-5p
4.20	3.56	1.85	1.73	1.54
ABCG2	FIS1	BMF	ABTB1	AGO2
AQP1	SMAD2	CD82	AHRR	AKT1
AQP4	ZEB1	FOXJ2	AKT1	ARID3A
AR		FOXO3	ALOX5	CAPNS1
ARF1		MAPK1	ANGPT2	CTDSPL
ARPP19		MTHFD1	APC	FGFR3
BANP		NSUN5	APLN	FKBP5
BMI1		PMAIP1	ARID3A	GSK3B
CDK6		RAN	ARID3B	HOXA1
CRKL		TUSC2	BAK1	IGF1R
CTNNB1			BBC3	MTMR3
ESRRG			BCL2	MTOR
FAS			BCL2L2	NOX4
FH			BCL3	RAVER2
FOXM1			BMF	SERPINE1
GNAI1			BMPR1B	SMARCA5
HMGB1			BTG2	TRIB2
HOXA10			CBFB	
HSPB6			CCNJ	
IGF1R			CD44	
ITGB3			CDH5	
KITLG			CDKN2A	
MAPK1			CDKN2D	
MCL1			CEBPA	
MTDH			CGN	
MYC			CSNK2A1	
NFATC3			CYP24A1	
NOD2			DGAT1	
NPR1			DKK3	
NRP1			DRAM2	
PBX3			DUSP6	
PDCD4			E2F2	
PICSAR			E2F3	
POLR3D			EGFR	
PTEN			EIF4EBP1	
RAB11A			EIF5A2	
RAB14			ENPEP	
RAC1			EPO	
RUNX2			EPOR	
SUZ12			ERBB2	
TAC1			ERBB3	
TFRC			ETS1	
TRPC5			Fas	
USP14			FES	
VDAC1			FGFR2	
VEGFA			FZD6	

VIM
YWHAZ

GAB2
GLI1
GRIN2A
GSS
HK2
HMGA1
HMGA2
HOTTIP
ICAM2
IGF1R
IGF2
IKZF2
IKZF3
IKZF4
IL6R
IRF4
JAK2
KLC2
KLF13
LACTB
LIFR
LIN28A
LIN28B
LIPA
MAN1B1
MAP3K11
MAPK14
MCL1
MEGF9
MMP13
MMP2
MMP26
MUC1
MXD1
NCOR2
NES
NEU1
NKIRAS2
NTRK3
PCTP
PHF8
PIAS3
PIGF
PIK3CB
PIK3CD
PODXL
PPP1CA
PRDM1
PRKRA
PTH1R

RAF1
RPS6KA1
SCNN1A
SEMA4C
SET
SFRP5
SGPL1
SIRT7
SMAD4
SMO
SPHK1
STARD13
STAT3
SUV39H1
TBC1D1
TET2
TNF
TNFAIP3
TP53
TP53INP1
VDR
VPS4B
VPS51

hsa-miR-145-5p	hsa-miR-19b-3p	hsa-miR-214-3p	hsa-miR-21-5p	hsa-miR-342-3p
1.49	1.49	1.45	1.09	1.07
ABCC1	ARID4B	ALPK2	ABCB1	BIRC6
ABHD17C	ATXN1	ARL2	AKT2	BMP7
ABRACL	BACE1	ASF1B	ANKRD46	CTBP2
ACTB	BCL2L11	ATF4	ANP32A	DNMT1
ADAM17	BCL3	BAX	APAF1	E2F1
ADD3	BMPR2	BCL2L11	BASP1	GEMIN4
AKR1B10	CUL5	BCL2L2	BCL10	ID4
ALDH3A1	CYP19A1	BIRC5	BCL2	IKBKG
ALPPL2	DNMT1	CADM1	BCL6	MTDH
ANGPT2	ESR1	CCL5	BMI1	SREBF1
AP1G1	GCM1	CD274	BMPR2	SREBF2
APH1A	HIPK1	CDK6	BTG2	TAB2
ARF6	HIPK3	CPD	CADM1	TAB3
ARL6IP5	KAT2B	CTNNB1	CASC2	
BNIP3	MTUS1	EZH2	CASP8	
BRAF	MXD1	FGFR1	CBX4	
C11orf65	MYCN	GALNT7	CCL20	
CAMK1D	MYLIP	GSR	CCR1	
CBFB	NCOA3	HDGF	CDC25A	
CCDC43	PITX1	ING4	CDK2AP1	
CD28	PKNOX1	JAG1	CEBPB	
CD40	PPP2R5E	LTF	CLU	
CD44	PRKAA1	LZTS1	COL4A1	
CDH2	PTEN	MAP2K3	COX2	
CDK4	SMAD4	MAPK1	DAXX	
CDK6	SOCS1	MAPK8	DDAH1	
CDKN1A	TGFBR2	MEF2C	DERL1	
CEP19	TLR2	NRAS	DNM1L	
CFTR	TNFAIP3	PAPPA	DOCK4	
CLINT1	TP53	PIM1	DOCK5	
COL5A1		PLXNB1	DOCK7	
CPEB4		POR	DUSP10	
CRNDE		POU4F2	E2F1	
CTGF		PSMD10	EGFR	
CTNND1		PTEN	EGLN1	
DDC		QKI	EIF4A2	
DDX17		RAB15	ERBB2	
DDX6		SEMA4D	FASLG	
DFFA		SRGAP1	FBXO11	
DTD1		SRGAP2	FMOD	
E2F3		SUFU	FOXO1	
EGFR		TP53	FZD6	
EIF4E		TWIST1	GAS5	
EPAS1		UBE2I	GDF5	
ERG		XBP1	HMGB1	
ESR1			HNRNPK	

ETS1
F11R
FAM3C
FAM45A
FLI1
FSCN1
FXN
FZD7
GMFB
GOLM1
HDAC11
HDAC2
HLTF
HMGA2
HOXA9
IFNB1
IGF1R
ILK
IRS1
IRS2
ITGB8
JADE1
JAG1
KLF4
KLF5
KREMEN1
LYPLA2
MAP2K6
MCM2
MDM2
MEST
MIXL1
MMP1
MMP12
MMP14
MSH3
MTDH
MTMR14
MUC1
MYC
MYO5A
MYO6
MYOCD
MYRF
NAIP
NANOG
NDRG2
NDUFA4
NEDD9
NFATC1
HPGD
ICAM1
ICOSLG
IGF1R
IL12A
IL1B
IRAK1
ISCU
JAG1
JMY
LRP6
LRRFIP1
MAP2K3
MARCKS
MEF2C
MSH2
MSH6
MSLN
MTAP
MYD88
NAV3
NCAPG
NCOA3
NFIA
NFIB
NTF3
OXTR
PCBP1
PCGF2
PDCD4
PIAS3
PIK3R1
PLAT
PLOD3
PPARA
PPIF
PSMD9
PTEN
PTPN14
PTX3
RASA1
RASGRP1
RECK
REST
RHO
RHOB
RPS7
RTN4
SATB1
SERPINB5

NIPSNAP1
NRAS
NUDT1
PAK4
PARP8
PIGF
PODXL
POU5F1
PPM1D
PPP3CA
PTP4A2
PXN
ROBO2
ROCK1
RPA1
RPS6KB1
RREB1
RTKN
SENP1
SERINC5
SERPINE1
SET
SMAD2
SMAD3
SOCS7
SOX2
SOX9
SP1
SP7
SPTBN1
SPTLC1
SRGAP1
STAT1
SWAP70
TGFB2
TGFBR2
TIRAP
TMEM9B
TMOD3
TNFSF13
TPM3
TPRG1
TSPAN6
TUG1
VEGFA
VPS51
YES1

SERPINI1
SETD2
SIRT2
SMAD7
SMARCA4
SMN1
SOCS1
SOCS6
SOD3
SOX2
SOX5
SP1
SPRY2
STAT3
STUB1
TAP1
TCF21
TGFB2
TGFBI
TGFBR2
TGFBR3
TGIF1
TIAM1
TICAM2
TIMP3
TLR3
TM9SF3
TNFAIP3
TNFRSF10B
TOPORS
TP53BP2
TP63
TPM1
TRAF7
UBE2N
VEGFA
VHL
WWP1
YOD1

hsa-miR-132-3p	hsa-miR-16-5p	hsa-miR-523-3p	hsa-miR-409-3p	hsa-miR-221-3p	hsa-miR-636
1.01	1.00	0.98	0.84	0.83	0.79
AGO2	ACVR2A		AKT1	ADAM1A	SLC25A5
ARHGAP32	ADORA2A		ANG	ADAMTS6	
BDNF	AKT3		CTNND1	ANXA1	
CCNA2	APP		ELF2	APAF1	
CCNB1	ARHGDIA		FGA	ARF4	
CDKN1A	ARL2		FGB	ARIH2	
CRK	AXIN2		FGG	ARNT	
EGFR	BACE1		FRAT1	ASZ1	
FOXO1	BCL2		GAB1	BBC3	
GDF5	BDNF		IFNG	BCL2L11	
HBEGF	BIRC5		MET	BECN1	
IRAK4	BMI1		MGMT	BMF	
JPT1	BRCA1		NLK	BNIP3	
KLHL11	CADM1		PHF10	BNIP3L	
MAPK1	CAPRIN1		RDX	BRAP	
MMP9	CCND1		RECK	CDKN1B	
MUC13	CCND2		RSU1	CDKN1C	
PIK3R3	CCND3		STAG2	CERS2	
RAF1	CCNE1		UGT2B17	CORO1A	
RASA1	CCNT2		ZEB1	CREBZF	
RB1	CDK6			CTCF	
SIRT1	CDS2			CXCL12	
SLC2A1	CHEK1			DDIT4	
SMAD2	CHUK			DICER1	
SOX4	CLDN2			DIRAS3	
SOX5	FGF2			DKK2	
SOX6	FGFR1			DVL2	
SPRED1	GLS2			ESR1	
SPRY1	HDGF			ETS1	
TJAP1	HGF			FMR1	
TLN2	HMGA1			FOS	
YY1AP1	HMGA2			FOXO3	
	IFNG			GJA1	
	IGF1R			GRB10	
	IL12B			HECTD2	
	KDR			HMGXB4	
	KRAS			HOXB5	
	MAP7			ICAM1	
	METTL13			KIT	
	MTOR			MBD2	
	MYB			MDM2	
	NCOR2			MEOX2	
	NCSTN			MGMT	
	OPRM1			MMP2	
	PIM1			MYBL1	
	PPM1D			NAIP	

PRDM4
PTGS2
PURA
RAF1
RECK
RICTOR
RPS6KB1
SLC6A4
SOCS3
SOX5
SOX6
TP53
TPPP3
UCA1
UNG
VEGFA
WEE1
WNT3A
WNT4
YAP1
ZYX

PAK1
PIK3R1
PTEN
RAB1A
RAD51
RB1
RECK
RUNX1
SELE
SIRT1
SOCS1
SOCS3
SSX2IP
STAT5A
STMN1
TBK1
TCEAL1
TICAM1
TIMP3
TMED7
TNFSF10
TP53
TRPS1
USP18
WEE1
ZEB2

hsa-let-7b-5p	hsa-miR-210-3p	hsa-miR-29a-3p	hsa-miR-30b-5p	hsa-miR-106a-5p	hsa-miR-17-5p
0.78	0.78	0.72	0.64	0.61	0.61
ACTG1	AIFM3	ABL1	ATG12	APC	ABCA1
ACVR1	ALDH5A1	ADAM12	BCL2	APP	ADAR
AGO1	ATG7	ADAMTS9	BCL6	ARID4B	APP
AKT2	BDNF	AHR	BCL9	ATG7	BCL2
ANAPC1	BNIP3	AKT2	BECN1	ATM	BCL2L11
CCNA1	BTK	AKT3	CAT	BCL10	BMP2
CCNA2	CASP8AP2	ALDH5A1	CCNE2	BMP2	BMPR2
CCND1	COL4A2	ATG9A	CTHRC1	CASP7	BRCA2
CCND2	CPEB2	BACE1	DLL4	CCND1	CCL1
CDC25A	DDAH1	BCL2	DNMT1	CDKN1A	CCND1
CDC34	DIMT1	BCL7A	EIF2S1	CDX2	CCND2
CDK6	E2F3	CACNA1C	EIF5A2	CXCL8	CDKN1A
COL3A1	EFNA3	CALCR	ERG	CYP19A1	CLOCK
CPEB1	EHD2	CCND1	HOXA1	E2F1	CLU
CPEB3	FGFRL1	CCND2	MBNL1	ERCC1	CYP7B1
CPEB4	FOXN3	CCNT2	MBNL2	FAS	DAPK3
CTHRC1	FOXP3	CD276	MBNL3	FASTK	DNAJC27
CYP2J2	GPD1L	CD93	NOTCH1	HIF1A	DNMT1
E2F2	HIF1A	CDC42	PDGFRB	HIPK3	E2F1
EZH2	HIF3A	CDC7	RASAL2	HMGA2	E2F3
HMGA1	HOXA1	CDK2	RUNX2	IL10	EGR2
HMGA2	HOXA9	CDK4	SERPINE1	LIMK1	EPAS1
HRAS	HSD17B1	CDK6	SIX1	MAPK9	ETV1
IFNB1	IGFBP3	CEACAM6	SMAD1	MFN2	FBXO31
IGF1R	INPP5A	CLDN1	SNAI1	MGST2	GPR137B
IGF2BP1	ISCU	COL10A1	SOCS1	MYB	HBP1
IGF2BP2	KCMF1	COL1A2	TP53	MYLIP	HIF1A
IRS2	LDHA	COL3A1		PTEN	HSPB2
LGR4	LDHB	COL4A1		RARB	ICAM1
LIN28A	MCM3	COL4A2		RB1	IGFBP3
LIN28B	MNT	COL5A2		RBL2	ITGB8
LRIG1	MRE11	CPEB3		RND3	JAK1
MTPN	NCAM1	CPEB4		RUNX1	KAT2B
NR2E1	NDUFA4	CYP2C19		RUNX3	LDLR
NRAS	NPTX1	DICER1		SIRPA	LIMK1
PDGFRA	P4HB	DKK1		SLC2A3	MAP3K12
PRDM1	PIM1	DNMT1		STAT3	MAPK9
RDH10	PLK1	DNMT3A		TGFBR2	MDM2
RPIA	PTBP3	DNMT3B		TIMP2	MEF2D
TGFBR1	PTPN1	ELN		ULK1	MFN2
TLR4	PTPN2	FBN1		VEGFA	MMP2
TNFRSF10B	RAD52	FGA			MYC
	SDHD	FGB			NABP1
	SH3BGRL	FGG			NCOA3
	STMN1	FOXO3			NPAS3
	TFRC	FSTL1			NPAT

THSD7A
TP53I11
TWIST1
VMP1
XIST
XPA

GLUL
GPR85
GSK3B
HBP1
HMGCR
IFNAR1
IGF1
IMPDH1
ITGA11
ITGA6
ITGB1
ITIH5
KDM5B
KEAP1
KLF4
KREMEN2
LAMC2
LOX
LPL
MCL1
MMP2
MUC1
MYC
MYCN
NASP
NAV3
NFIA
NMI
PDGFRB
PER1
PIK3R1
PPM1D
PPP1R13B
PTEN
PXDN
QKI
RAN
RASGRP1
RNASEL
ROBO1
S100B
SAPCD2
SERPINB9
SERPINH1
SETDB1
SFRP2
SLC22A7
SPARC
SRGAP2
TDG

PDLIM7
PHLPP1
PKD2
PKNOX1
PPP2R2A
PTEN
PTPRO
RAD21
RB1
RBL1
RBL2
RND3
RUNX1
SELE
SIRPA
SMAD4
SMURF1
SOCS6
STAT3
TBC1D2
TCEAL1
TCF3
TGFBR2
TIMP3
TLR7
TNF
TNFSF12
TP53COR1
TP53INP1
TRIM8
UBE2C
VEGFA
VLDLR
WEE1
YES1
ZBTB4
ZFYVE9
ZNFX1

TET1
TET2
TET3
TFEB
TNFAIP3
TRAF4
TRIM68
VDAC1
VEGFA
ZFP36

hsa-miR-30c-5p	hsa-miR-92a-3p	hsa-miR-483-5p	hsa-miR-20a-5p	hsa-miR-138-5p
0.58	0.52	0.52	0.38	0.38
BCL9	ARID4B	ALCAM	ABL2	ADGRA2
BECN1	BCL2L11	CKB	ANKH	AKT1
CAMK2D	BMPR2	FAM160B2	APP	ARHGEF3
CASP3	CCL8	MAPK3	ARHGAP12	BAG1
CCND2	CD69	NOTCH3	ATG16L1	BCL11A
CDC42	CDH1	RHOA	BAMBI	BLCAP
CTGF	CPEB2	SRF	BCL2	CASP3
DDIT4	DNMT1		BCL2L11	CCND1
DLL4	DUSP10		BMPR2	CCND3
DNMT1	ESR2		BNIP2	CD274
EIF2S1	FBXW7		CCND1	CDH1
FASN	HDAC2		CCND2	CYTOR
FOXO3	HIPK1		CDKN1A	EED
HDAC4	HIPK3		CRIM1	EID1
HSPA4	IKZF1		DAPK3	EIF4EBP1
IDH1	ITGA5		DNMT1	EZH2
IER2	KAT2B		DUSP2	FERMT2
IL11	KLF2		E2F1	FOSL1
JAK1	KLF4		E2F3	FOXC1
MCL1	LASP1		EGLN3	GNAI2
MTA1	MAP2K4		EGR2	H2AFX
MTTP	MAPK8		EPAS1	HIF1A
NCOR2	MAPRE1		ETV1	IGF1R
NOTCH1	MYCBP2		FBXO31	KDM5C
PAK1	MYLIP		GJA1	LCN2
RARB	NR1H4		HIF1A	MAP3K11
RASAL2	OSBPL2		IRF2	MXD1
RFX6	OSBPL8		ITGB8	NFKB1
RUNX2	PCGF5		KIF26B	PTK2
SERPINE1	PHLPP1		KIT	RARA
SMAD1	PTEN		LIMK1	RELN
SNAI1	RAD21		MAP2K3	RHOC
SNAI2	RFFL		MAP3K12	RMND5A
SOCS3	RGS5		MAP3K5	ROCK2
TGIF2	SIRT1		MAPK9	S100A1
TP53	SOCS5		MCL1	SENP1
TWF1	STAT3		MEF2D	SIRT1
UBE2I	TGFBR2		MYC	SLC45A3
VIM	TP63		NFKBIB	SNAI2
			NRAS	SOX4
			PHLPP2	SOX9
			PKD1	SUZ12
			PKNOX1	TERT
			PPARG	TWIST2
			PPP2R2A	VIM
			PRKG1	YAP1

PTEN	ZEB2
PTPRO	
PURA	
RB1	
RB1CC1	
RBL1	
RBL2	
REST	
RGS5	
RUNX1	
RUNX3	
SIRPA	
SMAD4	
SMAD7	
STAT3	
TCEAL1	
TGFBR1	
TGFBR2	
TIMP2	
TP53INP1	
TSG101	
UBE2C	
VEGFA	
WEE1	
ZFYVE9	

hsa-miR-193a-5p	hsa-miR-382-5p	hsa-miR-28-3p	hsa-miR-31-5p	hsa-miR-199a-3p
0.32	0.31	0.28	0.28	0.26
ERBB2	DRD1	STAT5B	ABCB9	AKT1
IGF2BP1	MXD1	TP53	ARID1A	APOE
ING5	NFIA		ARPC5	CAV2
MTOR	PTEN		BAP1	CD44
NLN	YBX1		C1QTNF9	CDK7
PIK3R3			CASR	DNAJA4
SRR			CDK1	FLT1
TFAP2A			CREG1	FOXA2
TP73			CXCL12	FUT4
WT1			DACT3	HGF
			DKK1	IGF1
			DMD	ITGA3
			DOCK1	KDR
			E2F2	MAPK1
			EMSY	MAPK14
			ETS1	MAPK8
			FOXO3	MAPK9
			FOXP3	MET
			FZD3	MTOR
			GNA13	PAK4
			HIF1AN	PTGS2
			HOXC13	SMARCA2
			ICAM1	STK11
			IL25	TFAM
			ITGA5	VEGFA
			JAZF1	YAP1
			KLF13	ZHX1
			LATS2	
			MAP4K4	
			MCM2	
			MET	
			MLH1	
			MMP16	
			MPRIP	
			NFAT5	
			NUMB	
			PPP2R2A	
			PRKCE	
			RAB27A	
			RASA1	
			RDX	
			RET	
			RHOA	
			RHOBTB1	
			SATB2	
			SELE	

SGPP2
SLC1A2
SMAD4
SOX4
SP7
SPRED1
SPRED2
SPRY1
SPRY3
SPRY4
SRC
STK40
STMN1
TBXA2R
TIAM1
WASF3
XRCC5
YY1

hsa-miR-376c-3p	hsa-miR-520e-3p	hsa-miR-29c-3p	hsa-let-7e-5p	hsa-miR-152-3p
0.17	0.17	0.16	0.16	0.15
ACVR1C	CD46	ADAM12	AGO1	ADAM17
BCL2	EGFR	AKT2	ARID3A	ALCAM
BMI1	MAP3K14	AKT3	AURKB	ATG14
GRB2	MAP4K4	BACE1	CCND1	CCKBR
IGF1R	PFKP	BCL2	EIF3J	CCND1
NR5A2	ZBTB7A	CCND2	EZH2	CD151
RUNX2		CD274	FASLG	CD274
TGFA		CDC42	HMGA2	CSF1
TGFBR1		CDK6	IGF1	DKK1
UGT2B15		CNOT6	IGF1R	DNMT1
UGT2B17		COL10A1	LIN28A	FGF2
		COL15A1	MMP9	FGFR3
		COL1A1	MPL	HLA-G
		COL1A2	MYCN	IGF1R
		COL21A1	PLK1	IRS1
		COL3A1	SMC1A	ITGA5
		COL4A1	TNFAIP3	KLF4
		COL4A2	TNFRSF10B	KRAS
		COL5A2	WNT1	MAFB
		COL7A1		NRP1
		CREB5		PIK3R3
		CTNND1		PTEN
		DNMT3A		TACC3
		DNMT3B		TGFA
		FBN1		WNT1
		FGA		XIST
		FGB		
		FGG		
		FRAT2		
		FZD4		
		FZD5		
		GAPDH		
		HMGCR		
		IGFBP1		
		ITGA6		
		ITGB1		
		KLF4		
		LAMC1		
		LAMC2		
		LOX		
		LRP6		
		MCL1		
		MMP15		
		MMP2		
		MMP24		
		MYCN		

NASP
PDGFRB
PER1
PHLDB2
PPP1R13B
PTEN
RCC2
RFX7
SERPINH1
SIRT1
SP1
SPARC
SRSF10
TARBP1
TDG
TET2
TFAP2C
TGIF2
TIAM1
VEGFA
WNT4

hsa-miR-30a-3p

0.15

BECN1
CDK6
CYR61
MECP2
NFATC3
NOTCH1
RUNX2
SLC7A6
THBS1
TMEM2
VEZT
XBP1
