# Supplementary material for: Tissue-Protective and Anti-Inflammatory Landmark of PRP-Treated Mesenchymal Stromal Cells Secretome for Osteoarthritis
Source: Int J Mol Sci. 2022 Dec 14;23(24):15908. doi: 10.3390/ijms232415908 (PMC9788137; doi:10.3390/ijms232415908)
Supplement: Supplementary file 1 [file ijms-23-15908-s001.zip › Table S7.pdf]

Table S7 – Univocal first quartile EV-miRNA targets

ABCA1

ABCB1

ABCB9

ABCC1

ABCG2

ABHD17C

ABL1

ABL2

ABRACL

ABTB1

ACTB

ACTG1

ACVR1

ACVR1B

ACVR1C

ACVR2A

ADAM12

ADAM17

ADAM1A

ADAMTS6

ADAMTS9

ADAR

ADD3

ADGRA2

ADORA2A

AGO1

AGO2

AGPAT2

AHR

AHRR

AIFM3

AKR1B10

AKR1C2

AKT1

AKT2

AKT3

ALCAM

ALDH3A1

ALDH5A1

ALOX5

ALPK2

ALPPL2

ANAPC1

ANG

ANGPT2

ANKH

ANKRD46

ANP32A

ANXA1

AP1G1

APAF1

APC

APH1A

APLN

APOE

APP

AQP1

AQP4

AR

ARF1

ARF4

ARF6

ARHGAP12

ARHGAP19

ARHGAP32

ARHGDIA

ARHGEF3

ARID1A

ARID3A

ARID3B

ARID4B

ARIH2

ARL2

ARL6IP5

ARNT

ARPC5

ARPP19

ASF1B

ASZ1

ATF4

ATG12

ATG14

ATG16L1

ATG4A

ATG7

ATG9A

ATM

ATXN1

AURKB

AXIN2

BACE1

BAG1

BAK1

BAMBI

BANP

BAP1

BASP1

BAX

BBC3

BCAR1

BCL10

BCL11A

BCL2

BCL2L11

BCL2L2

BCL3

BCL6

BCL7A

BCL9

BDNF

BECN1

BIRC5

BIRC6

BLCAP

BMF

BMI1

BMP2

BMP7

BMPR1B

BMPR2

BNIP2

BNIP3

BNIP3L

BRAF

BRAP

BRCA1

BRCA2

BTG2

BTK

C11orf65

C1QTNF9

CACNA1C

CADM1

CALCR

CAMK1D

CAMK2D

CAPNS1

CAPRIN1

CARD10

CASC2

CASP3

CASP7

CASP8

CASP8AP2

CASR

CAT

CAV2

CBFB

CBX4

CCDC43

CCKBR

CCL1

CCL20

CCL5

CCL8

CCNA1

CCNA2

CCNB1

CCND1

CCND2

CCND3

CCNE1

CCNE2

CCNJ

CCNT2

CCR1

CD151

CD274

CD276

CD28

CD40

CD44

CD46

CD69

CD82

CD93

CDC25A

CDC34

CDC42

CDC7

CDH1

CDH2

CDH5

CDK1

CDK2

CDK2AP1

CDK4

CDK6

CDK7

CDK9

CDKN1A

CDKN1B

CDKN1C

CDKN2A

CDKN2D

CDS2

CDX2

CEACAM6

CEBPA

CEBPB

CEP19

CERS2

CFTR

CGN

CHEK1

CHUK

CKB

CLDN1

CLDN2

CLINT1

CLOCK

CLTC

CLU

CNOT6

COL10A1

COL15A1

COL1A1

COL1A2

COL21A1

COL3A1

COL4A1

COL4A2

COL5A1

COL5A2

COL7A1

COPS5

CORO1A

COX2

CPD

CPEB1

CPEB2

CPEB3

CPEB4

CREB5

CREBZF

CREG1

CRIM1

CRK

CRKL

CRNDE

CSF1

CSNK2A1

CTBP2

CTCF

CTDSP2

CTDSPL

CTGF

CTHRC1

CTNNB1

CTNND1

CUL2

CUL5

CXCL12

CXCL8

CYP11B2

CYP19A1

CYP24A1

CYP2C19

CYP2J2

CYP7B1

CYR61

CYTOR

DACT3

DAPK3

DAXX

DDAH1

DDC

DDIT4

DDX17

DDX6

DEDD

DERL1

DFFA

DGAT1

DHFR

DHFRP1

DICER1

DIMT1

DIRAS3

DKK1

DKK2

DKK3

DLL4

DMD

DNAJA4

DNAJC27

DND1

DNM1L

DNMT1

DNMT3A

DNMT3B

DOCK1

DOCK4

DOCK5

DOCK7

DRAM2

DRD1

DTD1

DUSP10

DUSP2

DUSP6

DVL2

DYRK2

E2F1

E2F2

E2F3

EED

EFNA3

EGFR

EGLN1

EGLN3

EGR1

EGR2

EHD2

EID1

EIF2S1

EIF2S3

EIF3J

EIF4A2

EIF4E

EIF4EBP1

EIF5A2

ELF2

ELN

EMSY

ENPEP

EP300

EPAS1

EPO

EPOR

ERBB2

ERBB3

ERCC1

ERG

ESR1

ESR2

ESRRG

ETS1

ETV1

EZH2

F11R

FAF1

FAM160B2

FAM3C

FAM45A

FAS

FASLG

FASN

FASTK

FBN1

FBXO11

FBXO31

FBXW7

FEN1

FERMT2

FES

FGA

FGB

FGF11

FGF2

FGFR1

FGFR2

FGFR3

FGFRL1

FGG

FH

FIS1

FKBP5

FLI1

FLT1

FMOD

FMR1

FOS

FOSL1

FOXA2

FOXC1

FOXJ2

FOXM1

FOXN3

FOXO1

FOXO3

FOXP3

FRAT1

FRAT2

FSCN1

FSTL1

FURIN

FUT4

FXN

FZD3

FZD4

FZD5

FZD6

FZD7

GAB1

GAB2

GALNT7

GAPDH

GAS5

GATA3

GCM1

GDF5

GEMIN4

GJA1

GLI1

GLS2

GLUL

GMFB

GNA13

GNAI1

GNAI2

GNAI3

GOLM1

GPD1L

GPR137B

GPR85

GRB10

GRB2

GRIN2A

GSK3B

GSR

GSS

H2AFX

HBEGF

HBP1

HDAC11

HDAC2

HDAC4

HDGF

HECTD2

HGF

HIF1A

HIF1AN

HIF3A

HIPK1

HIPK3

HK2

HLA-G

HLTF

HMGA1

HMGA2

HMGB1

HMGCR

HMGXB4

HMOX1

HNF4A

HNRNPK

HOTTIP

HOXA1

HOXA10

HOXA9

HOXB5

HOXC13

HPGD

HRAS

HSD17B1

HSPA4

HSPB2

HSPB6

ICAM1

ICAM2

ICOSLG

ID4

IDH1

IER2

IFNAR1

IFNB1

IFNG

IFNR

IGF1

IGF1R

IGF2

IGF2BP1

IGF2BP2

IGFBP1

IGFBP3

IKBKG

IKZF1

IKZF2

IKZF3

IKZF4

IL10

IL11

IL12A

IL12B

IL1A

IL1B

IL25

IL4

IL6R

ILK

IMPDH1

ING4

ING5

INPP5A

INSIG1

IRAK1

IRAK4

IRF2

IRF4

IRS1

IRS2

ISCU

ITGA11

ITGA3

ITGA5

ITGA6

ITGB1

ITGB3

ITGB8

ITIH5

JADE1

JAG1

JAK1

JAK2

JAZF1

JMY

JPH2

JPT1

KAT2B

KCMF1

KDM5B

KDM5C

KDR

KEAP1

KIF26B

KIT

KITLG

KLC2

KLF13

KLF2

KLF4

KLF5

KLHL11

KRAS

KREMEN1

KREMEN2

LACTB

LAMC1

LAMC2

LASP1

LATS2

LCN2

LDHA

LDHB

LDLR

LGR4

LIFR

LIMK1

LIN28A

LIN28B

LIPA

LOX

LPL

LRIG1

LRP6

LRRC8A

LRRFIP1

LTF

LYPLA2

LZTS1

MAFB

MAN1B1

MAP2K3

MAP2K4

MAP2K6

MAP3K11

MAP3K12

MAP3K14

MAP3K5

MAP3K9

MAP4K4

MAP7

MAPK1

MAPK14

MAPK3

MAPK7

MAPK8

MAPK9

MAPRE1

MARCKS

MAX

MBD2

MBNL1

MBNL2

MBNL3

MCL1

MCM2

MCM3

MDM2

MDM4

MECP2

MEF2C

MEF2D

MEGF9

MEN1

MEOX2

MEST

MET

METTL13

MFN2

MGMT

MGST2

MIXL1

MLEC

MLH1

MMP1

MMP12

MMP13

MMP14

MMP15

MMP16

MMP2

MMP24

MMP26

MMP9

MNT

MPL

MPRIP

MRE11

MSH2

MSH3

MSH6

MSLN

MT1M

MTA1

MTAP

MTDH

MTHFD1

MTMR14

MTMR3

MTOR

MTPN

MTTP

MTUS1

MUC1

MUC13

MXD1

MXI1

MYB

MYBL1

MYC

MYCBP2

MYCN

MYD88

MYLIP

MYO5A

MYO6

MYOCD

MYRF

NABP1

NAIP

NANOG

NASP

NAV3

NCAM1

NCAN

NCAPG

NCOA3

NCOR2

NCSTN

NDRG2

NDST1

NDUFA4

NEDD9

NES

NEU1

NF1

NFAT5

NFATC1

NFATC3

NFIA

NFIB

NFKB1

NFKBIB

NIPSNAP1

NKIRAS2

NLK

NLN

NMI

NOD2

NOS3

NOTCH1

NOTCH2

NOTCH3

NOX4

NPAS3

NPAT

NPR1

NPTX1

NR1H4

NR2E1

NR5A2

NRAS

NRP1

NSUN5

NTF3

NTRK3

NUDT1

NUMB

OPRM1

OSBPL2

OSBPL8

OXTR

P4HB

PAK1

PAK4

PAPPA

PARP8

PBX3

PCBP1

PCGF2

PCGF5

PCNA

PCTP

PDCD4

PDGFRA

PDGFRB

PDLIM7

PER1

PFKP

PHF10

PHF8

PHLDB2

PHLPP1

PHLPP2

PIAS3

PICSAR

PIGF

PIK3CB

PIK3CD

PIK3R1

PIK3R3

PIM1

PITX1

PKD1

PKD2

PKNOX1

PLAT

PLAU

PLK1

PLOD3

PLXNB1

PLXNC1

PMAIP1

PODXL

POLD1

POLR3D

POR

POU4F2

POU5F1

PPARA

PPARG

PPIF

PPM1D

PPP1CA

PPP1R13B

PPP2R2A

PPP2R5E

PPP3CA

PRAP1

PRDM1

PRDM4

PRDX6

PRKAA1

PRKCE

PRKCH

PRKG1

PRKRA

PSAP

PSMD10

PSMD9

PTBP3

PTEN

PTGS2

PTH1R

PTK2

PTP4A2

PTPN1

PTPN14

PTPN2

PTPN9

PTPRF

PTPRO

PTX3

PURA

PXDN

PXN

QKI

RAB11A

RAB14

RAB15

RAB1A

RAB27A

RAC1

RAD21

RAD51

RAD52

RAF1

RAN

RARA

RARB

RASA1

RASAL2

RASGRP1

RAVER2

RB1

RB1CC1

RBL1

RBL2

RCC2

RDH10

RDX

RECK

REG4

RELN

REST

RET

RFFL

RFX6

RFX7

RGS5

RHO

RHOA

RHOB

RHOBTB1

RHOC

RICTOR

RMND5A

RNASEL

RND3

ROBO1

ROBO2

ROCK1

ROCK2

RPA1

RPIA

RPS6KA1

RPS6KA3

RPS6KB1

RPS7

RREB1

RSU1

RTKN

RTN4

RUNX1

RUNX2

RUNX3

RXRA

S100A1

S100A8

S100B

SAPCD2

SATB1

SATB2

SCNN1A

SDHD

SELE

SEMA4C

SEMA4D

SENP1

SERINC5

SERPINB5

SERPINB9

SERPINE1

SERPINH1

SERPINI1

SET

SETD2

SETDB1

SFRP2

SFRP5

SGPL1

SGPP2

SH3BGRL

SH3PXD2A

SHMT2

SIRPA

SIRT1

SIRT2

SIRT7

SIX1

SLC16A2

SLC1A2

SLC22A7

SLC25A5

SLC2A1

SLC2A3

SLC45A3

SLC6A4

SLC7A6

SMAD1

SMAD2

SMAD3

SMAD4

SMAD5

SMAD7

SMARCA2

SMARCA4

SMARCA5

SMC1A

SMN1

SMO

SMURF1

SNAI1

SNAI2

SOCS1

SOCS3

SOCS5

SOCS6

SOCS7

SOD2

SOD3

SOX2

SOX4

SOX5

SOX6

SOX9

SP1

SP7

SPARC

SPHK1

SPRED1

SPRED2

SPRY1

SPRY2

SPRY3

SPRY4

SPTBN1

SPTLC1

SRC

SREBF1

SREBF2

SRF

SRGAP1

SRGAP2

SRR

SRSF10

SSSCA1

SSX2IP

ST7L

STAG2

STARD13

STAT1

STAT3

STAT5A

STAT5B

STK11

STK40

STMN1

STUB1

STX16

SUFU

SUV39H1

SUZ12

SWAP70

TAB2

TAB3

TAC1

TACC3

TAP1

TARBP1

TBC1D1

TBC1D2

TBK1

TBXA2R

TCEAL1

TCF21

TCF3

TDG

TERT

TET1

TET2

TET3

TFAM

TFAP2A

TFAP2C

TFEB

TFRC

TGFA

TGFB1

TGFB2

TGFBI

TGFBR1

TGFBR2

TGFBR3

TGIF1

TGIF2

THBS1

THSD7A

TIAM1

TICAM1

TICAM2

TIMP2

TIMP3

TIRAP

TJAP1

TLN2

TLR2

TLR3

TLR4

TLR7

TM9SF3

TMC7

TMED7

TMEM2

TMEM92

TMEM9B

TMOD3

TNF

TNFAIP3

TNFRSF10B

TNFSF10

TNFSF12

TNFSF13

TNK2

TOPORS

TP53

TP53BP2

TP53COR1

TP53I11

TP53INP1

TP63

TP73

TPM1

TPM3

TPPP3

TPRG1

TRAF4

TRAF7

TRIB2

TRIB3

TRIM11

TRIM68

TRIM8

TRPC5

TRPS1

TSG101

TSPAN6

TUG1

TUSC2

TWF1

TWIST1

TWIST2

UBE2C

UBE2I

UBE2N

UCA1

UGT2B15

UGT2B17

ULK1

UNG

USP14

USP18

VDAC1

VDR

VEGFA

VEZT

VGLL4

VHL

VIM

VLDLR

VMP1

VPS4B

VPS51

WASF3

WEE1

WNT1

WNT3A

WNT4

WT1

WWP1

XBP1

XIAP

XIST

XPA

XRCC5

YAP1

YBX1

YBX3

YES1

YOD1

YWHAZ

YY1

YY1AP1

ZBTB4

ZBTB7A

ZEB1

ZEB2

ZFP36

ZFYVE9

ZHX1

ZNF217

ZNFX1

ZYX
